# Fibroblast-like cells in mesothelioma can derive from tumor cells

**DOI:** 10.1038/s41418-025-01639-9

**Published:** 2025-12-16

**Authors:** Jose M. Garcia-Manteiga, Eltjona Rrapaj, Francesca Caprioglio, Francesco De Marchis, Andrea Lamarca, Liam S. Colley, Angelo Carretta, Daniela Finocchiaro, Francesca Mercalli, Annamaria Molinario, Gianluigi Arrigoni, Renzo Boldorini, Massimo P. Crippa, Rosanna Mezzapelle, Marco E. Bianchi

**Affiliations:** 1 Center for Omics Sciences, IRCCS Ospedale San Raffaele, Milan, Italy; 2 Chromatin Dynamics Unit, Division of Genetics and Cell Biology, IRCCS Ospedale San Raffaele, Milan, Italy; 3 School of Medicine, Vita-Salute San Raffaele University, Milan, Italy; 4 Department of Thoracic Surgery, IRCCS San Raffaele Scientific Institute, Milan, Italy; 5 Department of Pathology, IRCCS Ospedale San Raffaele, Milan, Italy; 6 Department of Health Science, School of Medicine, University of Eastern Piedmont Amedeo Avogadro, Novara, Italy; 7 Present Address: Department of Neuroscience, Karolinska Institutet, Stockholm, 171 77 Sweden

**Keywords:** Cancer microenvironment, Tumour heterogeneity, Gene expression

## Abstract

Pleural mesothelioma (PM) is an aggressive cancer that originates from mesothelial cells lining the pleura. To identify the different cell types in mesothelioma and their relationships, we performed single-cell RNAseq analyses of non-malignant pleura biopsies, PM biopsies and PM patient-derived organoids. Gene expression profiles of mesothelial and mesothelioma cells are very similar, suggesting that mesothelioma cells retain most properties of mesothelial cells. Surprisingly, in PM patient-derived organoids mesothelioma cells can acquire a fibroblast-like gene expression profile. Indeed, in most of the original PM biopsies a fraction of cells within the cluster of cancer-associated fibroblasts (CAFs) appear derived from tumor cells, with which they share the same genomic rearrangements. We confirmed by immunohistochemistry, and thus at the protein level, that cancer-derived fibroblast-like cells (CDFs) express smooth muscle actin, as most CAFs do, but have lost the same tumor suppressor proteins as the cognate mesothelioma cells. We propose that mesothelioma cells can become CDFs because they retain the ability of mesothelial cells to differentiate into fibroblasts. CDFs are thus tumor cells with fibroblast-like gene expression associated to tumors, and fulfil the definition of CAFs. CAFs generally support tumor progression, and in most tumors derive from resident fibroblasts or circulating mesenchymal cells. Our finding that a subset of CAFs derive from tumor cells, at least in mesothelioma, challenges current understanding of CAF origin. We suggest that interfering with the mesothelioma-to-CAF transition might offer an avenue to moderate tumor progression and resistance to therapy.

## Introduction

Mesothelioma is a highly aggressive cancer arising from mesothelium, mostly of the pleura [1]. Mesothelioma has three main histological subtypes, epithelioid, sarcomatoid and biphasic, that are associated with different prognoses, which overall remain dismal [2]. The mutational burden of mesothelioma is low; mutations in tumor suppressor genes such as BRCA-1-associated protein (BAP1), merlin (NF2), p16/CDKN and p53 have been reported [3], but driver mutations in oncogenes are rarely found [4].

Pleural mesothelioma (PM) is the most common subtype of mesothelioma and is mainly caused by asbestos exposure [5]. Inflammation is associated to all stages of PM development. In particular, upon asbestos injury, the release of the alarmin High Mobility Group Box1 (HMGB1) in the extracellular space contributes to the recruitment of macrophages that initiate the inflammation driving MPM onset and progression [6, 7]. Both because inflammation is a complex response involving many cell types and their crosstalk, and because of the decade-long time needed for PM development after asbestos exposure, relevant and tractable in vitro models of PM are lacking. Indeed, the features at the gene expression level of PM tumor cells and their relation to the mesothelial cells of origin and to the tumor microenvironment (TME) have been scarcely studied so far; a first atlas of mesothelioma cells based on single-cell RNA sequencing (scRNA-seq) appeared only recently [8].

Mesothelial cells have an epithelial morphology (cuboidal and flat) and are arranged in a single layer –the mesothelium– lining the body cavities (chest, abdomen and tunica vaginalis) [9]. Mesothelial cells express many epithelial markers such as cytokeratins and cadherins; however, as they derive from the embryonic mesodermal layer, they also express mesenchymal markers like vimentin and desmin [10]. In general, mesothelial cells show a high grade of plasticity and can differentiate in vitro into adipocyte- and osteoblast-like cells [11]. Upon inflammatory stimuli mesothelial cells can change their morphology and function, and can differentiate to smooth muscle cells and fibroblasts [12] in a process dubbed mesothelial to mesenchymal transition (MMT) [13, 14]; MMT is related to Epithelial to Mesenchimal Transition (EMT), whereby epithelial cells lose their cell polarity and cell-cell adhesion and gain migratory and invasive properties characteristic of mesenchymal cells. Indeed, pancreatic ductal adenocarcinomas (PDAC) and peritoneal metastases of ovarian and colorectal carcinomas can induce the transition of peritoneal mesothelial cells to various populations of cancer-associated fibroblasts (CAFs) [15–17]. CAFs were thus of obvious interest in defining the TME of PM.

In most tumors, CAFs are cancer-associated fibroblast-like cells that derive from diverse populations of resident fibroblasts, from other stromal cell types or from bone marrow derived mesenchymal cells [15]. CAFs have features of fibroblasts, and in scRNAseq experiments their overall gene expression profile matches closely that of “classical” fibroblasts, including the expression of collagens and other matrix proteins. However, CAFs are diverse in different tumors and different subpopulations can be recognized even within the same tumor. In PM, the cross-talk between CAFs and PM cells is only partially explored [16]; however, CAFs are reported to instigate mesothelioma progression via secretion of a number of cytokines [17] and to influence adversely patient survival [18].

Our approach was to define first the gene expression signature of mesothelial cells, from which PM tumor cells originate, and then use such signature to compare mesothelial to mesothelioma cells in patient biopsies. We also set up patient-derived organoid (PDO) models of mesothelioma and non-malignant pleura, and studied the relation of the cells in the PDOs to the cells in patient biopsies. Together with recent scRNAseq studies, our work offers a comprehensive cell atlas of pleura and PM, highlighting intra- and inter-tumor heterogeneity. Notably, we found that many fibroblast-like cells in PDOs share copy number variations (CNVs) with PM cells, indicating a tumor origin. This was confirmed in patient biopsies, where a subset of fibroblast-like cells also derive from mesothelioma cells. Thus, our study reveals that in mesotheliomas tumor cells can differentiate into fibroblast-like cells, that we call Cancer-Derived Fibroblast-like cells (CDFs). CDFs express typical fibroblast genes, but carry the mutations and CNVs that define tumor cells; thus, they are mesothelioma cells that resemble fibroblast at the gene expression level. Since various populations of fibroblast-like cells within a tumor are collectively called CAFs, we argue that mesothelioma CAFs can include cells that derive from the tumor itself. This discovery challenges conventional understanding of CAF origin and highlights the need for investigation into the role of cancer-derived fibroblasts in mesothelioma pathogenesis and therapeutic targeting.

## Results

### Mesothelial cells and mesothelioma cells share similar transcriptional profiles

We started our investigation of mesothelioma cell identity and plasticity from the comparison of three samples of non-malignant pleura and 5 biopsies from treatment-naïve mesothelioma patients collected from the Thoracic Surgery Unit of San Raffaele Hospital.

The three non-malignant pleural biopsies were collected from patients with different conditions: one from a patient with pleurisy caused by heart failure, representative of inflamed and reactive mesothelium, one from a patient with lung adenocarcinoma (the pleura sample was distant from the tumor area), and one from a patient with pneumothorax. Macroscopic features of the samples and H&E staining are shown in Supplementary Fig. S1A. Of note, many inflammatory cells were adjacent to the monolayer of mesothelial cells in the sample from the pleurisy patient (Supplementary Fig. S1B).

**Fig. 1:**
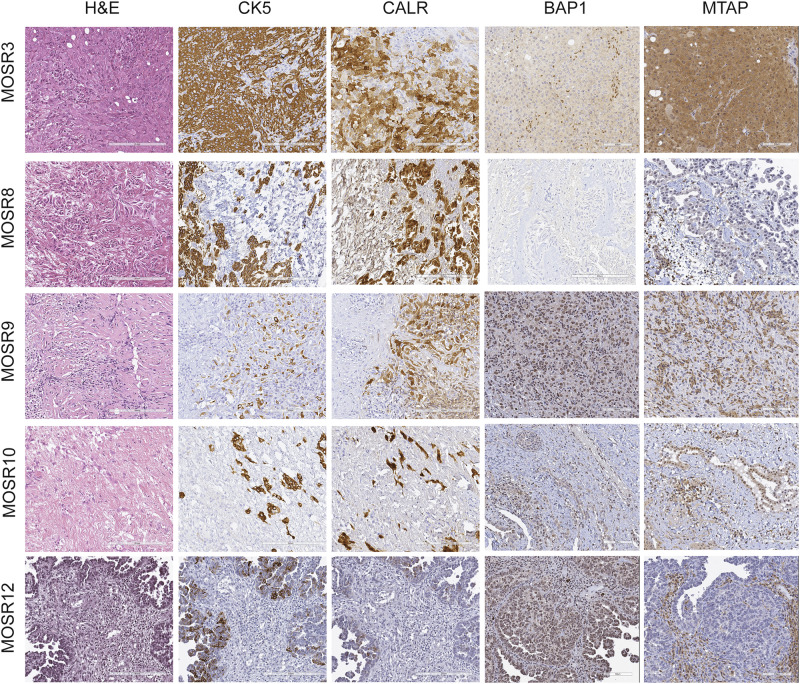
Immunohistochemistry of primary pleural mesothelioma biopsies. Sections from PM patients were stained with H&E. Immunohistochemistry for markers CK5, Calretinin (CALR), BAP1 and MTAP (among others) was used in all patients for the diagnosis of mesothelioma. Scale bar, 200 μm for H&E, CK5, CALR; 100 µm for BAP1 and MTAP.

The histological and clinical features of the PM patients and samples are described in Table 1; four samples showed an epithelioid histology, and one was biphasic (MOSR9). The diagnosis of mesothelioma was made by expert pathologists who evaluated 1) morphology 2) the invasion of cancer cell from the parietal pleura into the adipose tissue and 3) a panel of IHC markers. All biopsies were positive for CK5 and calretinin staining, two markers used to diagnose PM [19] (Fig. 1). BAP1, the most frequently mutated tumor suppressor gene in sporadic and familiar PM [20], was absent in three cases and present in two. CDKN2A is frequently lost in PM and CDKN2A homozygous deletion determined by fluorescence in situ hybridization (FISH) is diagnostic of malignancy in a mesothelial proliferation; recently, immunohistochemistry for methylthioadenosine phosphorylase (MTAP) has been adopted as a good surrogate of CDKN2A FISH [21]. In our cohort MTAP was lost in 3 cases out of 5.

**Table 1:** Clinical and pathological features of the first cohort of mesothelioma patients.

Patient	Genders	Age at diagnosis	Histotype	Asbestos exposure	CK 5	Calretinin	BAP1	MTAP	Survival (months)
MOSR3	M	76	Epithelioid	No	+	+	−	+	3
MOSR8	M	77	Epithelioid	Yes	+	+	−	−	NA
MOSR9	M	62	Biphasic	Yes	+	+	+	−	6
MOSR10	F	69	Epithelioid	Yes	+	+	−	+	35
MOSR12	M	81	Epithelioid	Yes	+	+	+	−	3
MOSR11	M	76	Epithelioid	NA	+	+	−	+	NA

**Fig. 2:**
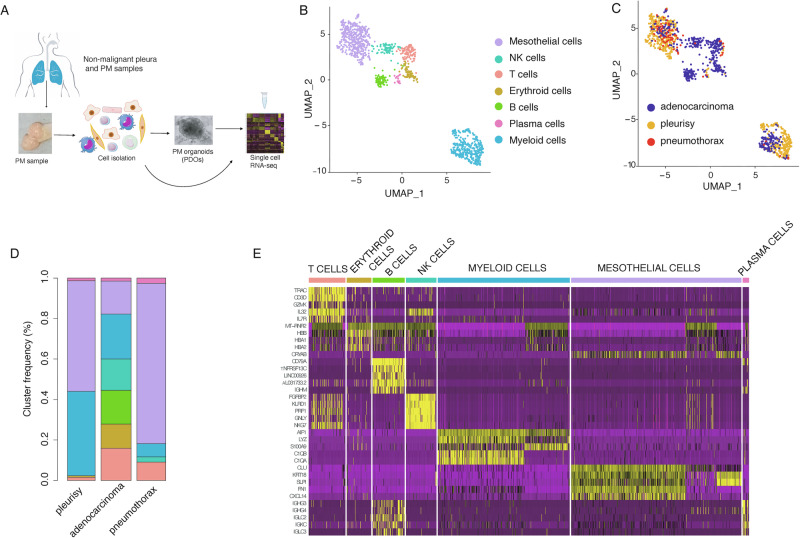
Single-cell expression profiling of non-malignant pleural specimens. **A** Study pipeline: samples obtained by video-assisted thoracic surgery were mechanically disaggregated to single cells. Viable cells were collected and processed by 10× Chromium Controller. **B** Uniform manifold approximation and projection (UMAP) of scRNA-seq data from *n* = 1 075 cells clustering in seven scRNA-seq groups, represented by different colors. **C** UMAP colored by sample origin. **D** Cell proportions in the 3 different samples of non-malignant pleura. **E** Heat map of expression of the top 5 marker genes for each of the 7 groups. Yellow: high expression; Purple: low/no expression. Each row represents a gene, and each column represents a single cell.

We used 10× Chromium droplet-based scRNAseq on the three non-malignant pleural samples to define the cellular components of pleura (Fig. 2A). After isolating vital and high-quality cells from all samples (see Methods, Supplementary Figs. S2–5 and Supplementary Tables 1, 2) we integrated cells form the 3 samples (*n* = 1075), identifying 7 main clusters of cells based on their specific cell markers (Fig. 2B–E and Supplementary Fig. S6, Supplementary Table 3). One cluster represented cells of mesothelial origin, characterized by the expression of cytokeratin 18 (KRT18) and mesothelin (MSLN); the other clusters represented myeloid cells (lysozyme, LYZ, and S100A9), B cells (CD79A), plasma cells (antibody J chain, JCHAIN), T cells (CD3D), NK cells (granulysin, GNLY) and erythroid cells (hemoglobin B, HBB). The pleurisy sample contained the largest myeloid infiltrate while the sample from the adenocarcinoma patient contained a high number of B, T and NK cells (Fig. 2B–D). The pneumothorax sample contained fewer cells than the former (83 vs ~600) but showed the highest proportion of mesothelial cells ( ~ 66%), with the rest representing myeloid, NK/T and erythroid cells (Fig. 2B–D).

Among the genes expressed by mesothelial cells, haptoglobin (HP) was highly represented, in contrast to its low representation in the other clusters (Supplementary Fig. S6I). Haptoglobin is a glycoprotein that binds free hemoglobin, preventing it from generating free oxygen species and thus exerting a deleterious oxidative activity. Haptoglobin is predominantly produced in the liver but has also been proposed as a potential biomarker for mesothelioma [22, 23].

**Fig. 3:**
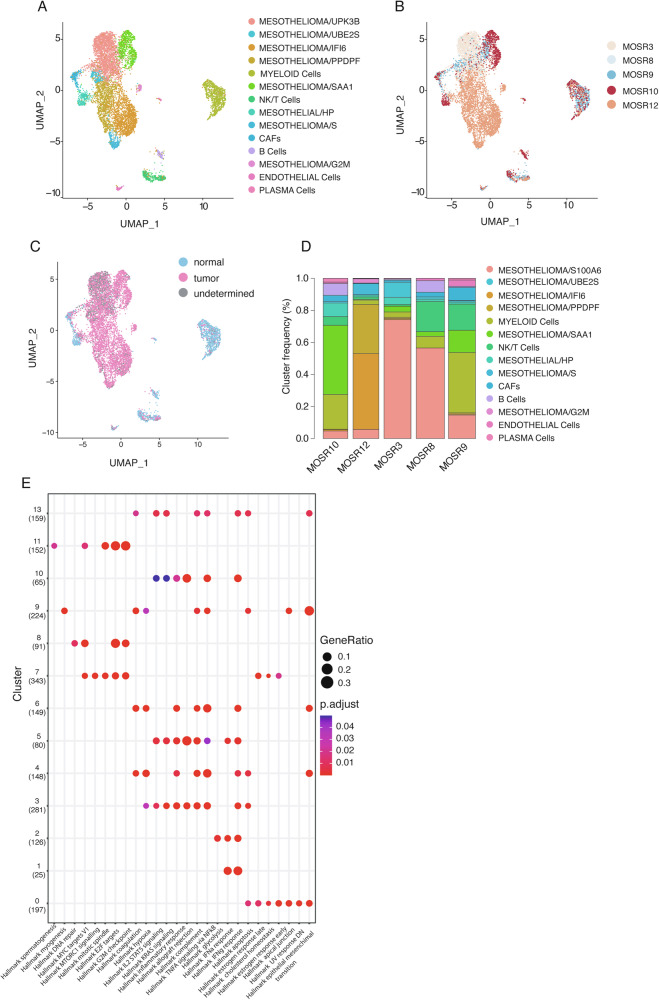
Single-cell expression profiling of human mesothelioma. **A** UMAP visualization of *n* = 8 005 cells (median nGene = 1153, nUMI = 3 472) clustering into 14 different populations. **B** Sample origins. **C** Normal or Tumor identification of cells based on scRNA-Seq CNV calls by Numbat. **D** Cell fractions in each of the 5 different samples. **E** Dot plot of the top 10 significant Hallmark Pathways enriched in specific clusters. The number of Differentially Expressed Genes in clusters (FDR < 0.05, log2FC > 1) is indicated under the name of the clusters.

We then examined single-cell data from the 5 PM biopsies and identified 14 main cell populations (Fig. 3A, B). Six clusters represented myeloid, plasma, B, NK/T and endothelial cells (the latter expressing CD37 and von Willebrand factor), and Cancer Associated Fibroblasts (CAFs) identified using as reference the scRNA seq data of fibroblasts from mesothelioma samples [8] (Supplementary Fig. S7A–E). Known mesothelioma markers (cytokeratins KRT8, -18 and -19; secretory leukocyte peptide inhibitor, SLPI) were expressed in each of the other eight cell clusters (Fig. 3A; Supplementary Fig. S8, Supplementary Table 4).

One cluster (mesothelial/HP) was made up of cells of mesothelial origin expressing the haptoglobin gene, like the mesothelial cells from non-malignant samples; we thus tested whether it comprised mesothelioma or mesothelial cells. To genetically differentiate tumor from normal cells, we used Numbat, an R package that detects the presence of copy number variations (CNV) from scRNAseq data by combining differential expression of contiguous genes with haplotype phasing [24]. Numbat identified as normal most of the stromal and immune cells from the microenvironment and as tumor most cells of mesothelial origin, except those in the mesothelial/HP cluster; we concluded that the mesothelial/HP cluster represents genetically normal mesothelial cells present in the tumor biopsies alongside the tumor cells (Fig. 3C, Supplementary Fig. S7F).

The mesothelial cells in the other 7 clusters contained CNVs and were scored as tumor (Supplementary Fig. S7F); we will henceforth refer to them as mesothelioma. They were differentiated by the expression of specific genes, some of them already identified as enriched in mesothelial/mesothelioma cells: Mesothelioma/UPK3B expressed the UPK3B gene (uroplakin); Mesothelioma/SAA1 expressed Serum Amyloid A1; Mesothelioma/UBE2S expressed Ubiquitin Conjugating Enzyme E2S. Remarkably, two clusters (Mesothelioma/IFI6 and Mesothelioma/PPDF) expressed genes related to IFN responses, while Mesothelioma/G2M and Mesothelioma/S expressed cell-cycle genes (Fig. 3E). Most patient biopsies contained cells from different mesothelioma clusters (Fig. 3B–D).

Notably, CAFs, mesothelial cells (mesothelial/HP) and 2 clusters of mesothelioma cells (Mesothelioma/SAA1 and Mesothelioma/UPK3B) were enriched in DEGs belonging to the Epitelial-to-Mesenchimal Transition (EMT) pathway (Fig. 3E).

Tumor cells were the most abundant in four out of five biopsies, while the fraction of myeloid cells and CAFs was very variable, and sometimes sizeable (Fig. 3D). Of note, tumor cells from different biopsies clustered in separate albeit adjoining populations, indicating that tumor cells were distinguishably different in different patients; in contrast, myeloid cells from different patients clustered together, as well as B, NK, T and plasma cells (Fig. 3B). In brief, this clustering pattern underscores the heterogeneity in mesothelioma cell populations as opposed to the relative homogeneity of immune cells from different patients.

**Fig. 4:**
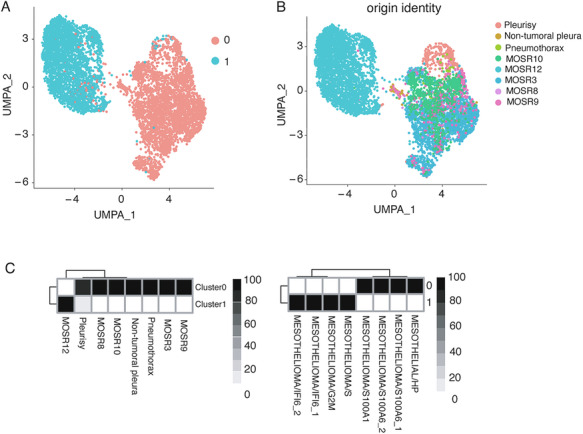
Comparison of malignant and non-malignant mesothelial cells. **A** UMAP representation of unbiased clusters in normal mesothelial cells and mesothelioma cells integrated from all biopsies. **B** Sample origins. **C** Percentage of cells of the different samples (left) and different mesothelioma and mesothelial clusters (right) within the two unbiased clusters in panels (**A**) and (**B**).

We next investigated the relationship between mesothelioma and mesothelial cells, excluding all other cells (Fig. 4). We represented the cells from the various samples in a new UMAP space by applying the anchoring procedure included in Seurat. Unsupervised analysis identified just 2 major clusters. Cluster 0 contained cells from non-malignant and mesothelioma samples, namely the cell populations identified as Mesothelioma/UPK3B, Mesothelioma/UBE2S, Mesothelioma/SAA1 and mesothelial/HP, in addition to most mesothelial cells from the non-malignant samples. Notably, mesothelioma and mesothelial cells are intermingled in Cluster 0 (Fig. 4A, B), suggesting that they overlap substantially in gene expression patterns. Cluster 1 mainly contained tumor cells from patient MOSR12 (Fig. 4A, Supplementary Table 5), but also cells from clusters Mesothelioma/IFI6, Mesothelioma/G2M, Mesothelioma/S and Mesothelioma/PPDPF (Fig. 4B, C). In cluster 1, differentially expressed genes are enriched in pathways of the IFN response and the cell cycle (Supplementary Fig. S9); these differences relate largely to inflammation and cell proliferation, and not to cell identity. Differential gene expression between mesothelial cells in cluster 0 and mesothelioma cells in the other clusters indicated that mesothelioma cells have increased expression of known mesothelioma markers (KRT19 and SAA1, including C15orf48 MOCCI [25]) and are enriched in mTOR signaling [26–28], oxidative phosphorylation and EMT pathways (Supplementary Fig. S10, Supplementary Table 6).

### Mesothelioma patient biopsies can be cultured as patient derived organoids

**Fig. 5:**
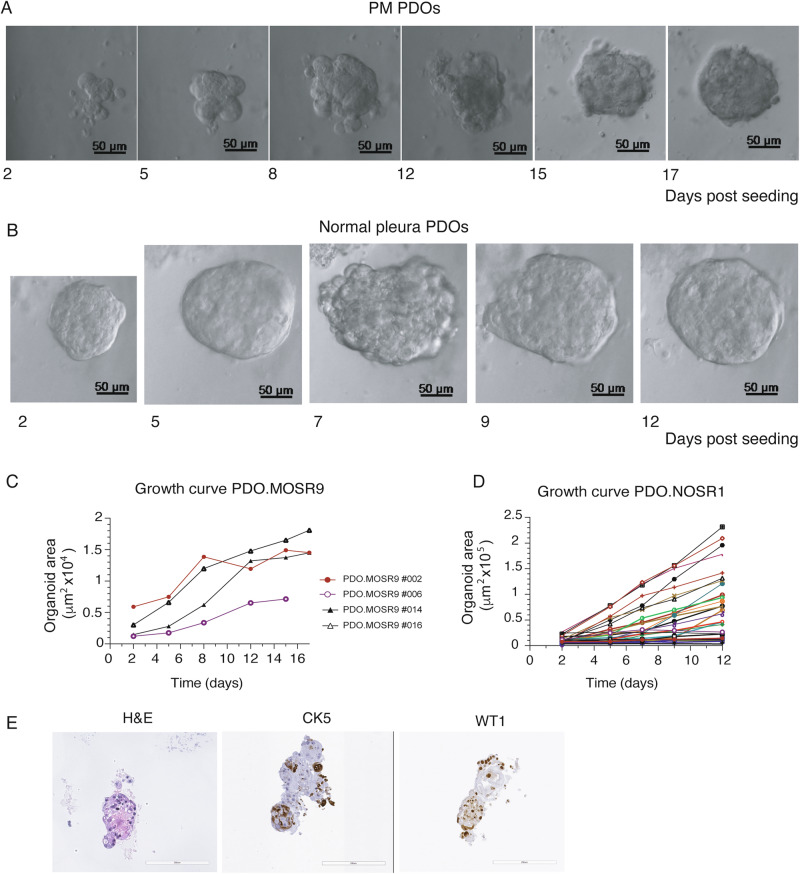
Characterization of organoids from non-malignant pleura and PM. **A** Representative bright-field images of PDOs derived from MPM biopsies. Organoid growth was monitored and measured at the indicated time. **B** Representative bright-field images of PDOs from a non-tumor pleura sample. **C** Growth curves of 4 PDOs from mesothelioma MOSR9. **D** Growth curves of 27 PDOs from non-tumor pleura NOSR1. **E** H&E and IHC staining for CK5 and WT1 of one representative MPM PDO. Scale bar, 200 µm.

Using the procedure described in the Methods section and summarized in Supplementary Fig. S11, we generated PDOs from tumoral and non-tumoral biopsies. All biopsies gave rise to organoids in Matrigel that grew for 3 weeks before needing re-isolation, fragmentation and re-culturing in Matrigel. We monitored daily organoid size and shape (Fig. 5A–D), observing an heterogenous growth rate; organoids from non-tumoral pleura transitioned from round aggregates to flat structures resembling the pleural lining (Fig. 5B). Histological and immunophenotypic analysis of formalin fixed tumoral PDOs showed that they were roundish in shape with an acellular matrix core (H&E staining; Fig. 5E) and are positive for the expression of CK5 and WT1.

**Fig. 6:**
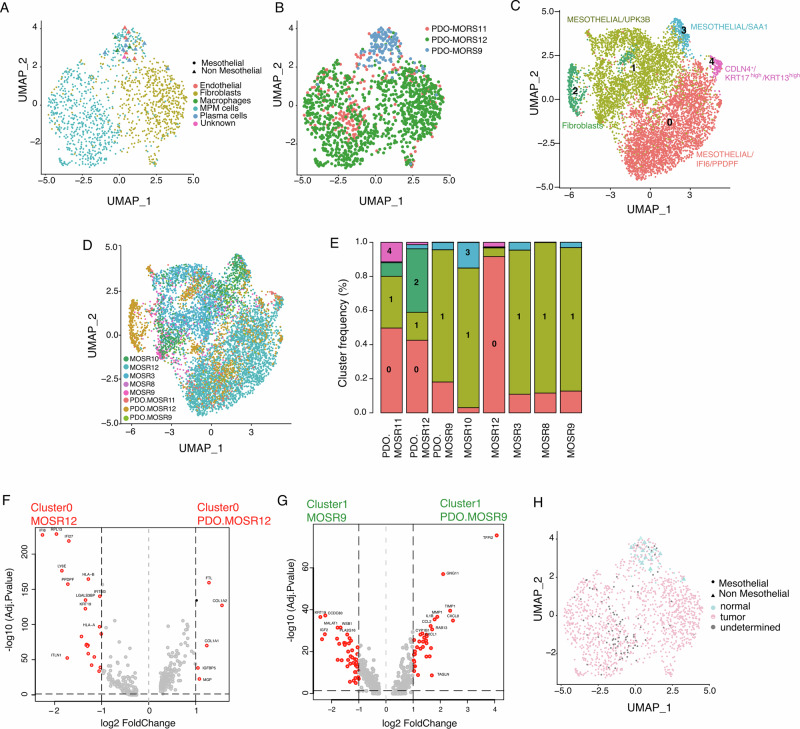
Identification of cell subpopulations in PDOs and comparison to PM cells from original biopsies. **A** UMAP visualization of PDO cells assigned using specific gene signatures and markers. **B** UMAP visualization of PDO cells highlighting sample origin. **C** UMAP plot of PM cells from patient biopsies and PDOs colored by unbiased clustering showing different transcriptional signatures. Clusters are numbered, and their signature is shown beside each cluster. **D** UMAP plot of PM cells colored by their sample of origin. **E** Cell subpopulation proportions of the different clusters in each of the samples. **F** Volcano plot of the DGE between cluster0 cells in tumor MOSR12 and cluster0 cells in PDO MORS12. **G** Volcano plot of the DGE between cluster1 in MOSR9 biopsy and PDOs. **H** Normal or Tumor identification of cells in biopsies based on scRNA-Seq CNV calls.

We then analyzed by scRNAseq the cells coming from 3 PM organoids (PDO.MOSR9, PDO.MOSR11 and PDO.MOSR12, filtered for vitality and highest sequencing quality) (Fig. 6, Supplementary Fig. S12). We identified 5 different cell populations (Fig. 6A); macrophages, plasma cells and endothelial cells (identified by their signature and specifically indicated by triangles) were present mainly in PDO.MOSR9 (Fig. 6A, B, Supplementary Fig. S12D–F), and overall were heavily depleted relative to the original biopsies.

Mesothelioma cells, identified using the transcriptomic signature defined in MM biopsies (Supplementary Table 2), represented about half of all cells and were present in all PDOs (Fig. 6A, Supplementary Fig S12B). Most of the remaining cells showed a fibroblastic signature, and they too were present in all PDOs (Fig. 6A; Supplementary Fig. S12C; Supplementary Table 7).

We first focused on identifying the differences between the tumor cells in the mesothelioma biopsies and those in the PDOs. To compare them, we overlayed mesothelial/mesothelioma cells from the tissue samples and the cells in the PDOs in the same transcriptomic space after applying batch correction and clustering. We identified five clusters (from 0 to 4) that reflected globally the same signatures found in the the biopsies, including mesothelioma cells with high epithelial to mesenchymal signature (EMT), plus one cluster comprising the organoid cells with the fibroblast signature (Fig. 6C, D; Supplementary Fig. S13; Supplementary Table 8).

In cluster 0, comprising mainly mesothelioma cells from the MOSR12 biopsy and the corresponding PDOs (Supplementary Table 9), tumor PDO cells downregulate relative to biopsy cells the expression of genes in the IFN response, MM markers such as KRT19 and ITLN1, and genes related to metabolism, immune system and antigen presentation, while they upregulate markers of fibrotic transformation and EMT, such as COL1A1, COL1A2, MGP and LUM (Fig. 6F; Supplementary Fig. S14A, B; Supplementary Tables 9, 10).

In cluster 1, comprising cells mainly from PDO.MOSR9 and its biopsy of origin, PDO cells upregulated genes involved in EMT (TFPI2, TIMP1) (Supplementary Fig. S14C), IL-1 regulation of extracellular matrix (CXCL6, IL1B, CCL2) and inflammatory response (IL6, CXCL8, CXCL1, CCL2) with a specific TNF-α/NF-κB signaling signature, while they downregulated cytokeratins (KRT19, KRT18, KRT7) (Supplementary Fig. S14C), TGFβ regulation of extracellular matrix (LUM, S100A10, TGFBI) and hypoxia (PTGIS, LDHA, NDRG1) (Fig. 6G; Supplementary Tables 9, 11).

We next focused on the cluster of PDO cells with fibroblast signature. These cells were in much higher proportion than in the original tumor biopsies, and we reasoned that they could derive either from an expansion of non-tumor fibroblasts present in the original tumor or from mesothelioma cells in the organoids. Using Numbat, we assessed the presence of CNVs in pooled PDO cells and confirmed the tumor origin of both mesothelioma and fibroblast-like cells. As a negative control, Numbat identified immune and stromal cells as non-malignant (Fig. 6H, Supplementary Fig. S12E). This indicates that mesothelioma cells in PDOs can adopt the gene expression signature of fibroblasts.

In brief, we could obtain and propagate PDOs from patient biopsies, and these retained the histological markers of the biopsies. scRNAseq highlighted a significant reduction of stromal and immune cell populations within the PDOs. The PDO cells with a mesothelial signature mirrored the transcriptomic signatures of mesothelioma cells in the original biopsy, but with a significant upregulation of fibrosis and EMT pathways and downregulation of the other pathways. Surprisingly, about half of the cells in the PDOs acquired a definite fibroblast signature even though they carry the CNVs of mesothelioma cells.

### scRNAseq of mesothelioma biopsies reveals that some tumor cells have a fibroblast-like gene expression profile

Having found that fibroblast-like cells in PDOs derived from mesothelioma cells, we wondered whether CAFs in the original PM biopsies were derived from normal fibroblasts or if they were tumor cells acquiring a fibroblast-like gene expression profile.

**Fig. 7:**
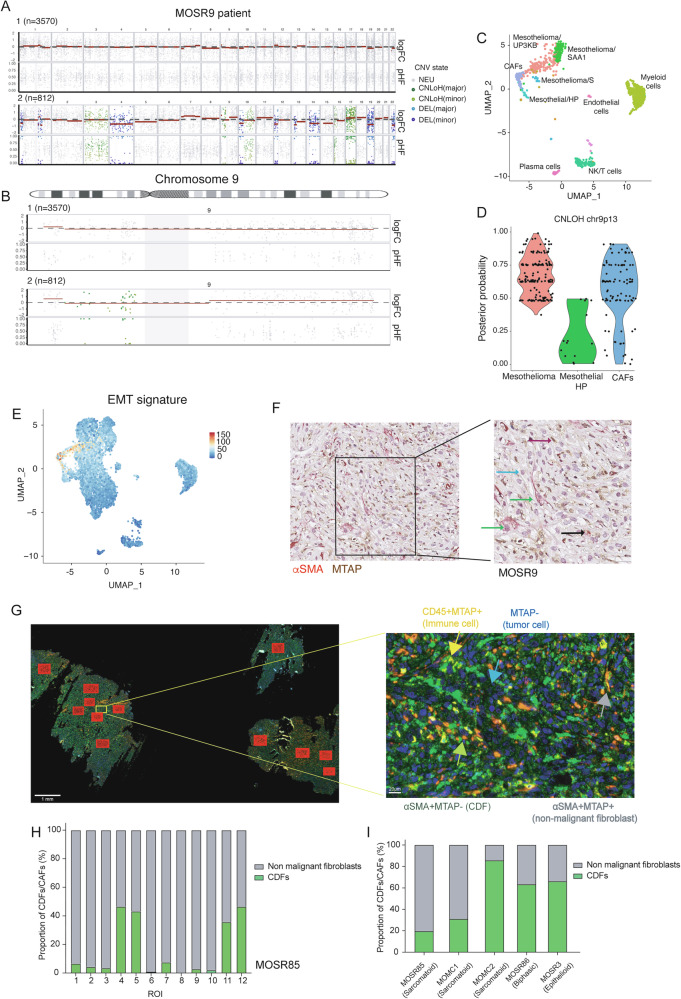
Single-cell CNV detection and multiplex immunohistochemistry reveal the existence of cancer-derived fibroblasts. **A** CNV profile of MOSR9 cells; log(FC) log expression fold-change, pHF parental haplotype frequency, NEU neutral, CNLoH copy-neutral Loss of Heterozigosity, DEL deletion, AMP amplification. Gray vertical bars represent centromeres and gap regions. The upper profile is of myeloid cells, the lower profile is of mesothelioma cells. **B** CNVs in chromosome 9 in MOSR9 normal (upper profile) and tumor cells (lower profile). The LoHs detected in these tumor cells involve the chr9p13 region, which encompasses the CDKN2A, CDKN2B and MTAP genes. **C** UMAP plot of MOSR9 cell types as in Fig. 3A. **D** Violin plot of posterior probabilities associated to individual cells from MOSR9, computed on the basis of chr9 LoHs. Each dot represents one cell. **E** UMAP plot of cells in Fig. 3A with expression of the EMT signature (Supplementary Table 2). **F** Double immunohistochemistry of MOSR9 biopsy. αSMA is red and cytoplasmic, MTAP is brown and mostly nuclear. The blue arrow points to one double-negative cell (mesothelioma tumor cell), the brown arrow to one αSMA-negative MTAP-positive cell (infiltrating immune cell), the black arrow to one double-positive cell (vascular smooth cell or pericyte) and the green arrows to two αSMA-positive MTAP-negative cells (tumor CAFs). **G** Multiplex immunohistochemistry of MOSR85 biopsy. Left: image scan with ROIs (red rectangles) used for quantification. MTAP is green, CD45 is yellow, αSMA is red and nuclei are blue. Scale bar: 1 mm. Right panel: detail of the selected ROI. A MTAP + CD45+ cell (immune cell) is indicated by the yellow arrow, a MTAP- cell (mesothelioma cell) by the blue arrow, a αSMA+ MTAP+ cell (non-tumor CAF) by the gray arrow and a αSMA+ MTAP- cell (CDF) by the green arrow. Scale bar: 20 μm. **H** Intra-patient variation of the percentage of CDFs within total CAFs. Bars represent different ROIs in the MOSR85 sample. **I** Inter-patient variation of the percentage of CDFs within total CAFs.

Tumor cells in biopsy MOSR9 had a CNV pattern (Fig. 7A) that included a copy-neutral Loss of Heterozygosity (CNLoH) in chromosome 9p13 spanning genes CDKN2A, CDKN2B and MTAP (Fig. 7B), which are frequently altered in PM. We then used this patient-specific chromosome 9 LoH to call normal and tumor cells in the original biopsy; Fig. 7C represents the cells deriving from MOSR9 in the same UMAP space as Fig. 3A. For each individual cell in the MOSR9 patient, an integrated probabilistic estimate can be computed of the presence of the chromosome 9-specific LoH (posterior probability); Fig. 7D shows that all mesothelioma cells have a high probability of bearing the LoH and mesothelial cells have a low probability; 28 CAFs have a low probability and 67 a high probability of bearing the LoH.

Tumor cells from biopsy MOSR12 and its cognate PDO shared a similar profile of CNVs (Supplementary Fig. S15A, B), confirming that PDO cells (including fibroblast-like cells) derive from tumor cells. From the scRNAseq data from the original biopsy, we determined that 7 out of 7 CAFs had the tumor CNVs.

Most CAFs (47 out of 49) in biopsy MOSR10 were scored as lacking CNVs. Fibroblast-like cells identified in biopsy MOSR3 mostly had tumor CNVs (24 out of 29). In the biopsy from patient MOSR8 we could identify two fibroblast-like cells, but none of them with tumor CNVs.

Notably, a trail of cells expressing the EMT signature appears to connect in the transcriptomic UMAP space the mesothelioma clusters to the CAF cluster (Fig. 7E, Supplementary Fig. 7B), suggesting that some CAFs derive from mesothelioma cells undergoing EMT.

To replicate our analysis, we examined a recently published set of scRNAseq data obtained from 13 PM patients (Table 2). Starting from the geneXcell matrix, we confirmed all the cell types originally identified in the 13 patients. In one of the patients (P10) no mesothelioma cells were detected (neither by the authors of the study nor by us), and this patient was considered non-informative. Three biopsies (patients P2, P6 and P9) did not contain identifiable fibroblast-like cells and one (P12) contained only 9; we did not reanalyze these scRNAseq data for the presence of CNVs. In the remaining 8 biopsies, we did detect tumor CNVs; in biopsy P11 Numbat actually recognized 2 different tumor clones, one that had lost BAP1 and one that had lost MTAP. Overall, of 12 informative patients, we found 6 with fibroblast-like cells with CNVs of the cognate tumor cells.

**Table 2:** Abundance of CAFs and CDFs in different patients in mesothelioma samples.

Patient ID	Sex	Histological type	BAP1 at diagnosis	BAP1 molecular status by Numbat	MTAP molecular status by Numbat	Number of tumor clones detected by Numbat	Total number of cells	Number of mesothelioma cells	Mesothelioma/total cell ratio	Number of CAFs	CAF/mesothelioma cell ratio	Number of CDFs	% of CDFs in total CAFs	Notes
scRNAseq, this study														
MOSR3	Male	Epithelioid	Lost	NA	NA	1	2282	2025	0.89	29	0.01	24	83	
MOSR8	Male	Epithelioid	Lost	NA	NA	1	69	42	0.61	2	0.05	0	0	there are too few cells for Numbat
MOSR9	Male	Biphasic	Retained	NA	NA	1	1172	363	0.31	95	0.26	67	70	
MOSR10	Female	Epithelioid	Lost	NA	NA	1	1270	634	0.50	49	0.08	2	4	
MOSR12	Male	Epithelioid	Retained	NA	NA	1	3212	3017	0.94	7	0.002	7	100	
scRNAseq, Giotti et al. 2024														
P1	Male	Sarcomatoid	Retained	NA	NA	1	10738	166	0.02	1662	10	25	2	
P2	Female	Epithelioid	Lost	NA	NA	NA	6131	5672	0.93	0	0	0	–	No fibroblasts
P3	Male	Biphasic	Lost	NA	NA	1	1564	38	0.02	31	0.8	0	0	
P4	Female	Epithelioid	Lost	NA	NA	2	13610	10926	0.80	325	0.03	29	9	
P5	Male	Epithelioid	Lost	NA	NA	1	7491	3359	0.45	138	0.04	0	0	
P6	Male	Epithelioid	NA	NA	NA	NA	8876	2280	0.26	0	0	0	–	No fibroblasts
P7	Male	Epithelioid	Lost	NA	NA	1	7755	295	0.04	199	0.7	4	2	
P8	Male	Epithelioid	Lost	NA	NA	3	4550	3976	0.87	43	0.01	2	5	
P9	Female	Epithelioid	Lost	NA	NA	NA	8496	207	0.02	0	0	0	-	No fibroblasts
P10	Male	Epithelioid	Lost	NA	NA	NA	4625	0	0.00	0	NA	NA	NA	no mesothelioma cells
P11	Male	Epithelioid	Retained	NA	NA	2	2720	1084	0.40	275	0.25	18	7	one clone BAP1-; 1 clone MTAP-
P12	Female	Biphasic	Retained	NA	NA	NA	2616	1143	0.44	9	0.008	NA	NA	few fibroblasts, not reanalyzed
P13	Female	Sarcomatoid	Retained	NA	NA	1	4816	116	0.02	613	5	110	18	

Patient ID	Sex	Histological type	BAP1 at diagnosis	BAP1 by IHC	MTAP by IHC	Number of cells in ROIs	Number of mesothelioma cells	Mesothelioma/total cell ratio	Number of CAFs	CAF/Mesothelioma cell ratio	Number of CDFs	% of CDFs in total CAFs
Multiplex IHC, this study												
MOMC1	Female	Sarcomatoid	pos		neg	494	232	0.47	203	0.88	63	31
MOMC2	Male	Sarcomatoid	pos		neg	941	835	0.89	195	0.23	167	86
MOSR3	Male	Epithelioid	neg	neg, focally		2396	1027	0.43	174	0.17	96	55
MOSR85	Male	Sarcomatoid	pos		neg	8123	4166	0.51	894	0.21	175	20
MOSR86	Female	Biphasic	pos		neg	8623	4426	0.51	2885	0.65	1827	63

*NA* not available.

Interestingly, the ratio of fibroblast-like cells to mesothelioma cells is extremely variable, from 0 to 10 (i.e. ten times more fibroblast-like cells than tumor cells); however, the 2 samples that contain more fibroblasts than tumor cells (P1, ratio of 10, and P13, ratio of 5) are both sarcomatoid, while in epithelioid and biphasic samples the ratios of fibroblast-like to tumor cells ranges from 0 to 0.8 in both our patients and Giotti’s [8] (Table 2).

In brief, we found fibroblast-like cells with tumor CNVs in 10 out of 17 informative mesothelioma patients belonging to two independent cohorts. Because these fibroblasts are not only cancer-associated, but appear to be cancer-derived, we will henceforth call them Cancer-Derived Fibroblast-like cells (CDFs).

### Protein-level analysis of mesothelioma biopsies confirms the existence of cancer-derived fibroblast-like cells

To further prove the presence within patient biopsies of tumor cells with fibroblast-like gene expression, we performed on MOSR biopsies a double immunohistochemistry staining for αSMA (present in activated fibroblasts, pericytes and smooth muscle cells) and for the tumor suppressor (TS) protein absent in the specific patient (BAP1 or MTAP as a proxy of CDKN2A/B).

In patient MOSR9, MTAP is absent in tumor cells, as seen in the IHC performed for diagnosis and the chromosome 9 LoH. In the double IHC for αSMA and MTAP (Fig. 7F) vascular cells (pericytes and smooth muscle cells) are double-positive for αSMA (red color, cytoplasmic) and MTAP (brown color, nuclear and cytoplasmic), mesothelioma cells are double-negative, infiltrating immune cells are positive for MTAP and negative for αSMA. CDFs were readily identifiable as negative for MTAP and positive for αSMA, confirming at the protein level the scRNAseq analysis.

In biopsies MOSR10 and MOSR3 we did detect by IHC rare cells that were negative for BAP1 and positive for αSMA (Supplementary Fig. S15C).

Thus, double IHC provides confirmation of the existence of CDFs in mesothelioma patients. However, it is difficult to obtain quantitative results from classical double IHC. We then analyzed archival biopsies of MOSR3 and 4 additional MP specimens collected from both Maggiore della Carità and San Raffaele Hospital (Supplementary Table 1).

We used two different multiplex IHC (mIHC) protocols using fluorescently labeled secondary antibodies to CD45, αSMA and MTAP or BAP1 (see Materials and Methods). The five mIHC stainings were comparable in quality and each enabled us to analyze several Regions of Interest (ROI) with high tumor burden (corresponding to the areas with more MTAP-negative or BAP1-negative cells) (Fig. 7G and Supplementary Fig. S16). The absolute number of CAFs (αSMA-positive cells) in MOSR3 ROIs was 174, of which the majority (*n* = 97) were CDFs (αSMA-positive BAP1-negative) (Table 2); the proportion of CDFs was fully in line with the scRNAseq data from the same patient. In the other four patients diagnosed with sarcomatoid or biphasic mesothelioma (MOSR85, MOSR86, MOMC1, MOMC2), the number of CDFs was substantive, ranging from 63 to 1827, and represented from 20 to 86% of cells expressing αSMA. CDFs also accounted for a variable but sizeable fraction (4–41%) of the tumor (TS-negative) cells in the four patients.

To investigate the extent of variation of CDF abundance in different areas of a single tumor, we compared each ROI in patient MOSR85 (Fig. 7G). We found high variability in different ROIs (Fig. 7H), suggesting that biopsy location in a tumor could by itself account for a high variability of CDF abundance from patient to patient. In any event, CDFs represented a sizeable proportion of all CAFs in every patient (Fig. 7I). We counted as CAFs all cells that were αSMA positive, but some of these might be pericytes or smooth muscle cells associated to vessels; on the other side, while most CAFs express αSMA, not all do. However, these uncertainties do not impact much on CAF and CDF prevalence in our samples.

In brief, using mIHC we did identify CDFs in all patients analyzed. CDFs were abundant and represented a variable but sizeable fraction of both tumor cells and CAFs; however, their distribution in different tumor areas is uneven.

## Discussion

The understanding of the cell composition of the tumor microenvironment is essential to elucidate the biology of a tumor and to find and optimize therapies. Pleural mesothelioma (PM) is a highly aggressive tumor and current treatments are not curative and improve 3-year survival rates for only a fraction of patients. Here, we report the identification in PM patients of fibroblast-like cells that share the same CNVs as cancer cells, that we named Cancer-Derived Fibroblast-like cells (CDFs). The fibroblast-like gene expression of CDFs was proven both by scRNAseq and by immunohistochemistry, and thus both at the RNA and protein level. CDFs co-cluster with classically defined CAFs in transcriptomic UMAP space, which is indicative of shared gene expression profiles; at the same time, they lack the same tumor suppressor proteins that are lost in cancer cells. Thus, CDFs are tumor cells with a fibroblast-like gene expression.

We started our analysis by identifying by scRNAseq mesothelial cells from non-malignant patients (one patient with pleurisy, one with lung adenocarcinoma, and one with pneumothorax). None of those samples is fully representative of normal pleura, but the very diversity of the medical conditions of these patients offered the opportunity to identify the commonalities among them. Overall, we found 12 357 cells that we could define as mesothelial based on the expression of cytokeratin 18 and mesothelin genes. The mesothelial cells from the 3 patients actually intermingled in the same cluster, which was clearly separated from the clusters of the various blood cells. Our identification of cell types and associated gene expression signatures in non-malignant pleuras broadly agree with those reported in a recent paper analyzing parietal pleural biopsies of pneumothorax patients [29]. Notably, we did not find any cluster for endothelial cells; correspondingly, the blood cells in the various samples do not appear to derive from blood circulating within blood vessels but rather represent abundant inflammatory infiltrate. Such blood cells may be absent or nearly absent from truly normal pleura from healthy patients. Likewise, we did not identify a cluster representing fibroblasts; we did find cells expressing collagens, but the same cells also expressed mesothelial markers.

We then analyzed by scRNAseq 5 biopsies deriving from PM patients. The transcriptomic pattern observed in mesothelial cells in non-malignant pleuras was instrumental in identifying mesothelial and mesothelioma cells in tumor samples, which were clearly separated from a cluster of CAFs (Fig. 3). Mesothelioma cells shared with mesothelial cells the same markers (MSLN, CXCL14, SLPI, ILTN1 and KRT18), as would be expected if mesothelioma cells derive from mesothelial cells. Indeed, markers of mesothelioma are also markers of mesothelial cells, and the difference between normal and tumor samples is mainly due to the number of cells expressing these markers: normal pleuras contain few mesothelial cells arranged in a monocellular layer and mesothelioma biopsies contain extensive 3D masses of mesothelioma cells.

Notably, the mesothelioma cells from different patients were different in abundance, possibly depending on the variable tumor burden in the biopsy, and clustered in transcriptomic UMAP space somewhat differently from each other, consistent with the notion that each patient might have a different constellation of mutations and epigenetic events driving tumoral (de)differentiation and tumor cell identity. Interestingly, mesothelial and mesothelioma cells distributed in 9 clusters. One of the clusters, which we called mesothelial/HP because of the expression of the haptoglobin gene (HP), comprises mesothelial cells from MM and non-malignant patients. The bioinformatic tool Numbat confirmed their identity of normal mesothelial cells by the absence of copy-number variations (CNVs). Thus, Mesothelial/HP cells are normal mesothelial cells even if they come from a MM patient.

When considering only mesothelial and mesothelioma cells we found two clusters: one cluster comprised both mesothelial and mesothelioma cells with several different signatures; the other comprised mainly mesothelioma cells with the interferon (IFN) signature. These results further underscore that mesothelioma and mesothelial cells are very similar in gene expression; this is not necessarily the case for the relation of tumor cells (i.e. in anaplastic carcinomas or high-grade serous ovarian carcinoma, HGSOC [30]) to their cells of origin. Tellingly, cells from the same patient distribute to different clusters, consistent with the possibility that subclones might be present, or that the TME is locally different in the same biopsy.

In our analyses, the most limiting factor was the physical size of the biopsy and therefore the number of recoverable cells. For this reason, and to obtain reliable in vitro models of PM, we established patient-derived organoids (PDOs) from biopsies of PM and non-tumor patients by adapting a protocol for the growth of prostate-derived organoids [31]. All samples gave rise to PDOs that grew for 3 weeks in Matrigel before needing re-isolation and re-culturing. When analyzed by scRNAseq, PDOs appeared depleted in inflammatory cells and enriched in mesothelioma and fibroblast-like cells. In principle, the fibroblast like-cells could derive either from CAFs or from the cancer cells present in the original biopsy. Interestingly, both mesothelioma and fibroblast-like cells in the same organoid showed the same pattern of CNVs; cells that have the same genome rearrangements are considered to have same clonal origin.

Most importantly, we could demonstrate that the original biopsies from which the PDOs derive also contain tumor cells that have a fibroblast-like gene expression profile. Since these fibroblast-like cells have tumor CNVs, they must derive clonally from mesothelioma cells in the same patient. Diverse populations of fibroblast-like cells within a tumor are collectively called CAFs, and by this measure CDFs are a subset of CAFs. It is important to note that our definition of CAFs does not depend on morphological features or the expression of a single marker protein, but from clustering of data points representing the global transcriptome of individual cells after dimensionality reduction with Uniform Manifold Approximation and Projection (UMAP). The co-clustering in scRNAseq of CDFs and other CAFs might to some extent depend on dimensionality reduction performed in UMAP; however, we think this unlikely because the difference between mesothelial cells and mesothelioma cells is clearly apparent at the same level of clustering resolution. Importantly, we confirmed the existence of CDFs, i.e. of cells clustering with CAFs but with tumor CNVs, in a recently published atlas of mesothelioma cells and their TME [8].

We further substantiated the existence of CDFs by classical double histochemistry: in biopsies from the mesothelioma patients analyzed by scRNAseq we found cells expressing αSMA (present in activated fibroblasts including most CAFs, pericytes and smooth muscle cells) and lacking MTAP or BAP1 (absent in tumor cells of specific patients). Multiplex IHC also identified a large number of cells fulfilling our operational definition of CDFs (αSMA-positive, TS-negative) in one patient previously analyzed by scRNAseq and four additional patients. Notably, in sarcomatoid patients three cell types (tumor cells, non-tumor CAFs and CDFs) have a spindle-shaped morphology.

Thus, two orthogonal approaches at the RNA and protein level allow us to conclude that mesothelioma samples taken from patients contain CAFs that derive from mesothelioma cells and not from classical fibroblasts or mesenchymal cells. The prevalence of CAFs, and of CDFs among CAFs, is widely variable among patients and even in different regions of a biopsy from a single patient. However, CDFs were present in substantial numbers in most patients of the 3 PM histotypes –epitheliod, biphasic and sarcomatoid. Mesothelioma is a rare cancer, and a specific effort to recruit a larger number of patients will be required to determine whether CAFs and CDFs are significantly more prevalent in sarcomatoid as opposed to epithelioid patients.

In most tumors, CAFs derive from diverse populations of resident fibroblasts, from other stromal cell types or from bone marrow derived cells [15]. To our knowledge, this is the first time that a fraction of CAFs in tumor patients have been shown to derive from the malignant cells in the tumor. While transitions from normal to tumor cells are conceptually obliged and have been identified in numerous studies, we show here that tumor cells can adopt an “almost normal” cellular identity. In principle, it is possible that driver genetic and epigenetic events first occur in fibroblasts and then fibroblasts become tumor cells, but this appears unlikely and in conflict with the finding that gene expression in mesothelioma cells resembles that of mesothelial cells, not of fibroblasts. Indeed, tumors arising from fibroblast-like cells are classified as sarcomas, and while sarcomas occasionally appear in pleura, these are rare events, while we find CDFs in most PM patients.

Possibly, CDFs had not been recognized so far because most pipelines for scRNAseq analysis of the TME classify any cell with CNVs as tumor, irrespective of their gene expression profile. However, we favor the idea that CDFs exist in PM because i) mesothelioma cells have a gene expression profile very similar to that of mesothelial cells, and ii) mesothelial cells can transdifferentiate into CAFs, for example in pancreatic cancer and in peritoneal metastases of ovarian and colorectal carcinomas [32–34]. In peritoneal metastases of intestinal adenocarcinomas, mesothelial cells become CAFs via Mesothelial-to-Mesenchimal Transition (MMT) [35]. The derivation of CAFs from adenocarcinoma cells was explicitly excluded in this study [35] based on the absence of simultaneous staining for adenocarcinoma and fibroblast protein markers, but these CAFs might have lost the expression of adenocarcinoma proteins while retaining the mutations and genomic rearrangements of adenocarcinoma cells.

In conclusion, we have compared by single-cell RNA sequencing the identity of cells in samples of non-malignant pleura, confirmed mesotheliomas and patient-derived organoids. We show that mesothelioma cells can differentiate within the tumor into CAFs, or more specifically into Cancer-Derived Fibroblast-like cells. From a practical point of view, we expect the occurrence and quantitative prevalence of CDFs to affect the prognosis of mesothelioma patients, and perhaps their response to treatments, but this must be proven by future clinical studies.

## Methods

### Patient samples

We collected 9 pleural mesothelioma (PM) samples and 3 samples of non-malignant pleura from the Thoracic Surgery units of IRCCS Ospedale San Raffaele and Maggiore della Carita’ hospital; 3 samples of normal pleura were obtained from pleurisy, pneumothorax, and adenocarcinoma patients. The use of all human samples was approved by the ethical committee of IRCCS Ospedale San Raffaele. All patients provided informed consent prior to sample acquisition.

### Generation and culture of human pleural and mesothelioma organoids

For the establishment of patient derived organoids fresh mesothelioma biopsies were collected, immediately frozen in 90% FCS, 10% DMSO and stored in liquid nitrogen. Biopsies were then thawed and minced using scalpels into small pieces (1–5 mm^3^). The small tissue fragments were collected and mashed through a 70 μm cell strainer until cell aggregates were obtained. If necessary, red blood cells were removed using a red blood lysis buffer (eBioscence). After washing and filtering though the 70 µm mesh, cell aggregates generated by mechanical disaggregation were embedded in melted Matrigel® Growth Factor Reduced basement membrane matrix, phenol red-free, 10 mL (#356231, Corning) and plated into ultra-low attachment bottom 24-well plates (Corning Costar CLS3473). The culture plates with just-seeded cell aggregates enclosed in matrigel were placed at 37°C in a 5% CO_2_ incubator for 20–30 min to allow Matrigel solidification. The medium contained adDMEM/F12 (#12634-034, Life technologies) supplemented with HEPES (#15630-056, Life technologies), L-Glutamine, Pen/Strep and 10 µM Y-27632 dihydrochloride, plus B27 supplement (#17504-044, Life technologies, Gibco), N2 supplement (#17502, Life technologies), 100 ng/ml Noggin (#120-10 C, Peprotech), 50 ng/ml rhEGF (#AF-100-15, Peprotech), 10 ng/ml of recombinant human basic FGF (#100-18B, Peprotech), 10% R-Spondin conditioned medium, and 50% Wnt conditioned medium. We refreshed the medium every 2–3 days. We monitored organoid growth daily and we acquired images with an inverted microscope (Axio Observer.Z1, Zeiss) every 2 days post-seeding. The organoids were subcultured after 3 weeks by using the harvesting solution of Trevigen (#3700-100-01, Cultrex). The final pellet of cell aggregates was resuspended in Matrigel and replated, splitting each organoid culture at a 1:2–1:4 ratio (the variability of ratio splitting is due to the quantity of organoids obtained and growth after day 0 of plating). Again, we replaced the medium every 2–3 days of culture. The workflow for the development of organoid cultures is summarized in Supplementary Fig. S2.

### Formalin-fixed, paraffin-embedded blocks of PM organoids

The PM organoids maintained in culture were collected after washing 2–3 times with ice-cold HBSS, scraping the Matrigel, pipetting up and down using cold 1000 μl tips and incubating for 1 h at 4°C to remove Matrigel from organoids. The organoid pellets obtained were washed, resuspended and fixed in 4% formalin for 90 min at room temperature. After formalin removal the pellet was washed once with PBS; the supernatant was removed and replaced with 10 ml of 80% ethanol with eosin. Eosin (a red dye) is added to allow the detection of the pellet during the embedding process and when cutting the blocks. We prepared a 3% agarose solution in PBS for embedding; the agar was melted and kept warm in the microwave and gently added (200–300 μl) with gentle flicking of the tube to fully resuspend the organoids. The tube containing the organoids in agar was kept for 15–20 min at −20°C to harden. The pellet-containing agar was removed and placed in IHC cassettes that were immersed in 80% ethanol overnight. The samples were processed and paraffin embedded according to standard cytological protocols of Anatomic Pathology Unit, Novara Hospital. Three-μm slices were used for IHC staining of mesothelioma markers and 5-μm slices for hematoxylin and eosin staining.

### Immunohistochemistry

Sections of formalin-fixed, paraffin embedded organoids and tissues were used for histopathological analysis. Hematoxylin and Eosin staining and Sirius Red staining were performed according to a standard protocol. For immunohistochemistry, rabbit monoclonal antibodies against calretinin (Abcam, ab92341, dilution 1:100), vimentin (EPR3776, abcam ab92547, dilution 1:200), WT-1 (abcam, ab216646, dilution 1:100), mesothelin (abcam, ab93620, dilution 1:50), mouse monoclonal cytokeratin 5/6 (D5/16 B4 abcam, ab 17133), MTAP (abcam ab126760, dilution 1:100) and rabbit polyclonal BAP-1 (abcam, ab199396) were used. Different antigen retrieval buffers were used to perform manual IHC staining according to manufacturers’ protocols.

### Multiplex IHC (mIHC)

mIHC was performed on 3 μm thick formalin-fixed, paraffin-embedded (FFPE) sections from PM samples (3 sarcomatoid, 1 biphasic and 1 epithelioid), using two different protocols.

#### Akoya protocol

Following deparaffinization and rehydration, antigen retrieval was carried out by heating the slides for 40 min at 95°C in ER2 buffer (BOND Epitope Retrieval Solution 2, Leica). Primary antibody incubation was conducted for 30 min at 36°C using a panel of four antibodies against MTAP (42-T, Santa Cruz, dilution 1:100), CD45 (X16/99, Leica, ready-to-use) and αSMA (ASM-1, Leica, ready-to-use). Nuclei were counterstained with DAPI. Image acquisition was performed using the Akoya Vectra Polaris imaging system (Imager 2.0). Slides were scanned at 40× magnification.

#### SignalStar protocol

Following deparaffinization and rehydration, antigen retrieval was carried out in boiling EDTA solution (#14747, Cell Signaling) for 5 min. Slides were first incubated with anti-MTAP antibody (clone E5R1I, #74683) O/N at 4 C, followed by incubation for 40 min at room temperature with 150 µL of SignalStar Imaging Round 1 for antibodies against αSMA (clone D4K9N + CO-0024-594, #25040) and CD45 (clone D9M8I + CO-0013-750, #56180). For MTAP staining we used complementary oligo #0055-647. After slides were rinsed in TBST for 30 s, tissues were fixed in 10% neutral buffered formalin for 5 min, rinsed in distilled water, and subjected to eight sequential rounds of amplification using Amplification Solutions 1 and 2 (each applied for 8 min and followed by a distilled water rinse). Ligation was performed by incubating the slides in 150 µL of SignalStar Ligation Buffer for 20 min at room temperature, followed by a rinse in distilled water. Nuclei were counterstained using DAPI. Slides were mounted with ProLong Gold Antifade Reagent (Cell Signaling #9071). Images were acquired using a SP8 laser scanning confocal microscope (Leica).

#### mIHC image analysis

Individual projects were created in QuPath for each tissue section. Regions of interest (ROI) were selected while visualizing solely the DAPI channel to prevent bias. Cell segmentation based on the DAPI channel was carried out in all selected ROIs using the “cell detection” function in QuPath. Subsequently, positivity thresholding was set for all markers of interest (MTAP, αSMA, and CD45). To minimize the number of false positives and negatives, the MTAP threshold was set based on MTAP-positive regions identified by classical IHC. Alternatively, the MTAP threshold was set so that all CD45-positive cells were also positive for MTAP.

The selected thresholds were maintained for all images analyzed, which was ensured by importing the same classifications into the project for each individual image. Once classifications had been set, we quantified the following: 1) Total number of cells per ROI (nuclei stained with DAPI), 2) Total number of MTAP+ cells, 3) Total number of αSMA+ cells, 4) Total number of CD45+ cells, 5) Number of MTAP+ αSMA− CD45− cells, 6) Number of αSMA+ MTAP− CD45− cells, 7) Number of MTAP+ αSMA+ CD45− cells, and 8) Number of MTAP+ αSMA+ CD45+ cells. Cancer-derived fibroblasts (CDFs) were defined as αSMA+ MTAP− CD45− cells, normal CAFs were defined as MTAP+ αSMA+ CD45− cells, and tumor cells were defined as the total number of cells minus MTAP+ cells.

### Preparation of single cell suspensions from mesothelioma biopsies and non-malignant pleura

The PM and non-malignant pleura biopsies were thawed and minced mechanically into small pieces of 1 cm^2^ using scalpels in sterile conditions. Further tissue dissociation into single-cell suspension was performed using Multi Tissue Dissociation Kit 2 (#130-110-203) combining mechanical with enzymatic dissociation, according to the manufacturer´s datasheet. GentleMACS Dissociators were used for the mechanical dissociation steps. After dissociation, each sample was applied to a filter supplied in the kit to remove any remaining larger particles from the single-cell suspension. Cells were finally suspended in 100 µl of PBS containing 0.04% BSA in 1.5 ml tubes and processed immediately for scRNAseq; cell density was 400 cells/µl for a total cell number of 3000 cells.

### Preparation of single cell suspension from patient derived organoids

The organoid medium was removed and the Matrigel plug was washed for 3 times with cold HBSS. Matrigel was dissolved using the harvesting solution of Trevigen Culturex following the manufacturer’s protocol. The organoid pellet was transferred into 15 ml tubes on ice and centrifuged to remove the remaining Matrigel. Organoid pellets were dissociated into single-cell suspensions by pipetting up and down several times with P100 tips; the cell suspension was checking under the microscope and filtered with 40 μm filters if necessary. Finally, the cell suspension was transferred to 1.5 ml tubes and processed as indicated for the preparation of single-cell suspensions from biopsies.

### Library construction using the GemCode platform

Cell suspensions of 10,000 cells from 8 patients and 5 patient derived organoids were prepared and vitality was assessed for each individual scRNA-seq experiment. Approximately 5000 cells were loaded onto a single-cell chip for GEM generation using the 10x Genomics Chromium Controller (10× Genomics, Pleasanton, CA). 3′mRNA-seq gene expression libraries were constructed using the Chromium Single Cell 3′ Library & Gel Bead Kit v2 (10× Genomics) according to the manufacturer’s guidelines. Briefly, cell suspensions were diluted in PBS with 0.04% BSA to a final concentration of 1×10^6^ cells/mL. Five thousand cells were loaded into each chip well to obtain a target output of 2 500 cells per sample. All reactions were performed in a C1000 Touch Thermal Cycler (Bio-Rad Laboratories, Hercules, CA). Twelve cycles were used for cDNA amplification, while the number of total cycles for the sample index PCR was calculated based on the cDNA concentration. Amplified cDNA and final libraries were evaluated using a Tape Station 4200 (Agilent Technologies, Santa Clara, CA) with a high sensitivity chip. scRNAseq libraries were sequenced on an Illumina Nextseq 500 with the standard sequencing protocol of R_1_ 26; I_1_ 8; I_2_ 0; R_2_ 98 nt read length to obtain 100,000 reads per cell.

Single cell encapsulation, cDNA amplification, library preparation and sequencing were performed by COSR (Center for Omic Sciences) at IRCCS Ospedale San Raffaele, and by IGATech.

### scRNA-Seq analyses

Single cell gene-count matrices were obtained with CellRanger (v6) and analyzed in R with Seurat. Briefly, matrices of single samples were filtered using quality parameters, i.e. cells with less than 200 genes expressed and genes expressed in less than 5 cells were excluded. Each sample dataset was further filtered by applying sample-specific filters regarding maximum number of genes per cell and mitochondrial percentage. After preprocessing steps with NormalizationData, FindVariablesFeatures and ScaleData regressing out nFeatures and percentage of mitochondrial genes, a variable number of principal components was chosen based on the ElbowPlot and clustering, and UMAP was performed to qualitatively evaluate single samples prior to integration. Unsupervised clustering using the Leiden algorithm was performed with a resolution level of 0.8 and UMAP clustering was used for 2-D frame viewing of clusters. The whole dataset contained 12,357 single cells at a median read depth of 56,000 reads/cell). Dead and low-quality cells were filtered out from each sample (Supplementary Figs. S2–5, Supplementary Tables 1, 2).

Integration of datasets was performed using FindIntegrationAnchors and IntegratedData Seurat functions with default parameters. Gene specific markers for clusters were obtained with FindAllMarkers function applying with a Wilcoxon rank-sum test on genes expressed in at least 10% of cells of either cluster and only.pos=T parameter. The Differential Gene Expression of genes between clusters and between samples was performed using FindMarkers function with the same parameters but with only.pos=F parameter to obtain also downregulated differentially expressed genes. Both Differentially Expressed Genes and specific cluster markers analyses were performed on RNA assay of scaled data.

EnrichR web based was used to identify pathways (MSigDB_hallmark_2020) significantly enriched (adjusted *P* < 0.05) using cluster specific marker genes. Genes with an adjusted *P* <  0.05 were considered significant. An additional absolute log(fold change) > 1 or 0.75 was used to consider significantly differentially expressed genes between clusters and/or samples. The identification of Cancer Associated Fibroblasts in our 5 biopsies integrated datasets (Supplementary Fig. 7B) was performed by using data in Giotti et al. [8] and the label transfer procedure in Seurat Package.

For the re-analysis of Giotti’s dataset (GSE190597), we downloaded and re-analyze with CellRanger the raw fastq files and used the same Seurat analysis as for our samples to identify all the different cell types present in the TME. GeneXcell matrices were used to identify both mesothelioma and fibroblastic cells before the CNV identification.

### CNV identification

Tumor vs Normal identification was performed by detecting copy number variations (CNVs) in single cells using the R package Numbat [24]. Numbat is a computational tool used to detect CNVs starting from single-cell RNAseq data; it combines several types of information to infer which cells carry genomic alterations typical of cancer. Specifically, Numbat detects expression changes of contiguous genes across the genome, as these can be due to amplifications or deletions of DNA segments. It also looks at the expression balance between maternal and paternal alleles at heterozygous sites, since shifts in this balance can indicate a CNV or a loss of heterozygosity. To increase sensitivity and reliability, Numbat uses haplotype information from population genetics data to link alleles across longer genomic stretches. Numbat first groups cells with similar expression and allele patterns and then identifies shared CNVs at the group level. Finally, it refines the analysis by assigning CNV profiles to individual cells.

In our analysis, the gene-cell integer UMI count matrix from Cell Ranger was given as input to the run_numbat function and the pileup_and_phase.R script provided with the package. Numbat identifies tumor cells by combining evidence from all CNVs; it then derives the aneuploidy probability for each cell to distinguish tumor versus normal cells.

### Statistics

Differentially expressed genes between clusters were identified by applying a Mann–Whitney non parametrical test using FindMarkers function in Seurat package. For functional enrichment with Enrichr, the *p*-value is calculated with the Fisher’s exact test, The *q*-value is an adjusted *p*-value calculated using the Benjamini–Hochberg method [36].

## Supplementary information

Supplementary figures

Data set 1-8

Data set 9

Data set 10

Data set 11

Table mIHC 1

## Data Availability

Single-cell data have been deposited at the NCBI GEO data repository under accession number GSE253973.

## References

[1] Boutin C, Schlesser M, Frenay C, Astoul P. Malignant pleural mesothelioma. Eur Respir J. 1998;12:972–81. 9817178 10.1183/09031936.98.12040972

[2] WHO Classification of Tumours, Thoracic Tumours, 5th edn. International Agency for Research on Cancer; 2020.

[3] Bott M, Brevet M, Taylor BS, Shimizu S, Ito T, Wang L, et al. The nuclear deubiquitinase BAP1 is commonly inactivated by somatic mutations and 3p21.1 losses in malignant pleural mesothelioma. Nat Genet. 2011;43:668–72. 21642991 10.1038/ng.855 PMC4643098

[4] Mezzapelle R, Miglio U, Rena O, Paganotti A, Allegrini S, Antona J, et al. Mutation analysis of the EGFR gene and downstream signalling pathway in histologic samples of malignant pleural mesothelioma. Br J Cancer. 2013;108:1743–9. 23558893 10.1038/bjc.2013.130 PMC3668472

[5] Robinson BM. Malignant pleural mesothelioma: an epidemiological perspective. Ann Cardiothorac Surg. 2012;1:491–6. 23977542 10.3978/j.issn.2225-319X.2012.11.04 PMC3741803

[6] Yang H, Rivera Z, Jube S, Nasu M, Bertino P, Goparaju C, et al. Programmed necrosis induced by asbestos in human mesothelial cells causes high-mobility group box 1 protein release and resultant inflammation. Proc Natl Acad Sci USA. 2010;107:12611–6. 20616036 10.1073/pnas.1006542107 PMC2906549

[7] Suarez JS, Novelli F, Goto K, Ehara M, Steele M, Kim JH, et al. HMGB1 released by mesothelial cells drives the development of asbestos-induced mesothelioma. Proc Natl Acad Sci USA. 2023;120:e2307999120. 37729199 10.1073/pnas.2307999120 PMC10523480

[8] Giotti B, Dolasia K, Zhao W, Cai P, Sweeney R, Merritt E, et al. Single-Cell View of Tumor Microenvironment Gradients in Pleural Mesothelioma. Cancer Discov. 2024;14:2262–78. 38959428 10.1158/2159-8290.CD-23-0017 PMC13109001

[9] Mutsaers SE, Wilkosz S. Structure and function of mesothelial cells. In: Peritoneal Carcinomatosis. Springer US: Boston, MA, pp 1–19. 10.1007/978-0-387-48993-3_1 17633044

[10] Batra H, Antony VB. The pleural mesothelium in development and disease. Front Physiol. 2014;284:eColletion 2014. 10.3389/fphys.2014.00284 PMC4117979 25136318

[11] Lansley SM, Searles RG, Hoi A, Thomas C, Moneta H, Herrick SE, et al. Mesothelial cell differentiation into osteoblast- and adipocyte-like cells. J Cell Mol Med. 2011;15:2095–105. 21070599 10.1111/j.1582-4934.2010.01212.x PMC4394220

[12] Liu Y, Dong Z, Liu H, Zhu J, Liu F, Chen G. Transition of Mesothelial Cell to Fibroblast in Peritoneal Dialysis: EMT, Stem Cell or Bystander?. Perit Dial Int. 2015;35:14–25. 25700459 10.3747/pdi.2014.00188 PMC4335923

[13] Mutsaers SE, Birnie K, Lansley S, Herrick SE, Lim C-B, Prele CM. Mesothelial cells in tissue repair and fibrosis. Front Pharm. 2015;6:113 eCollection 2015. 10.3389/fphar.2015.00113 PMC4460327 26106328

[14] Yang AH, Chen JY, Lin JK. Myofibroblastic conversion of mesothelial cells. Kidney Int. 2003;63:1530–9. 12631370 10.1046/j.1523-1755.2003.00861.x

[15] Yang D, Liu J, Qian H, Zhuang Q. Cancer-associated fibroblasts: from basic science to anticancer therapy. Exp Mol Med. 2023;55:1322–32. 37394578 10.1038/s12276-023-01013-0 PMC10394065

[16] Chrisochoidou Y, Roy R, Farahmand P, Gonzalez G, Doig J, Krasny L, et al. Crosstalk with lung fibroblasts shapes the growth and therapeutic response of mesothelioma cells. Cell Death Dis. 2023;14:725. 37938546 10.1038/s41419-023-06240-x PMC10632403

[17] Li Q, Wang W, Yamada T, Matsumoto K, Sakai K, Bando Y, et al. Pleural Mesothelioma Instigates Tumor-Associated Fibroblasts to Promote Progression via a Malignant Cytokine Network. Am J Pathol. 2011;179:1483–93. 21763682 10.1016/j.ajpath.2011.05.060 PMC3157262

[18] Borchert S, Mathilakathu A, Nath A, Wessolly M, Mairinger E, Kreidt D, et al. Cancer-Associated Fibroblasts Influence Survival in Pleural Mesothelioma: Digital Gene Expression Analysis and Supervised Machine Learning Model. Int J Mol Sci. 2023;24:12426. 37569808 10.3390/ijms241512426 PMC10419996

[19] Cury PM, Butcher DN, Fisher C, Corrin B, Nicholson AG. Value of the Mesothelium-Associated Antibodies Thrombomodulin, Cytokeratin 5/6, Calretinin, and CD44H in Distinguishing Epithelioid Pleural Mesothelioma from Adenocarcinoma Metastatic to the Pleura. Mod Pathol. 2000;13:107–12. 10697265 10.1038/modpathol.3880018

[20] Testa JR, Cheung M, Pei J, Below JE, Tan Y, Sementino E, et al. Germline BAP1 mutations predispose to malignant mesothelioma. Nat Genet. 2011;43:1022–5. 21874000 10.1038/ng.912 PMC3184199

[21] Chapel DB, Schulte JJ, Berg K, Churg A, Dacic S, Fitzpatrick C, et al. MTAP immunohistochemistry is an accurate and reproducible surrogate for CDKN2A fluorescence in situ hybridization in diagnosis of malignant pleural mesothelioma. Mod Pathol. 2020;33:245–54. 31231127 10.1038/s41379-019-0310-0

[22] López-Ríos F, Chuai S, Flores R, Shimizu S, Ohno T, Wakahara K, et al. Global Gene Expression Profiling of Pleural Mesotheliomas: Overexpression of Aurora Kinases and P16/CDKN2A Deletion as Prognostic Factors and Critical Evaluation of Microarray-Based Prognostic Prediction. Cancer Res. 2006;66:2970–9. 16540645 10.1158/0008-5472.CAN-05-3907

[23] Palstrøm NB, Overgaard M, Licht P, Beck HC. Identification of Highly Sensitive Pleural Effusion Protein Biomarkers for Malignant Pleural Mesothelioma by Affinity-Based Quantitative Proteomics. Cancers. 2023;15:641. 36765599 10.3390/cancers15030641 PMC9913626

[24] Gao T, Soldatov R, Sarkar H, Kurkiewicz A, Biederstedt E, Loh P-R, et al. Haplotype-aware analysis of somatic copy number variations from single-cell transcriptomes. Nat Biotechnol. 2023;41:417–26. 36163550 10.1038/s41587-022-01468-y PMC10289836

[25] Lee CQE, Kerouanton B, Chothani S, Zhang S, Chen Y, Mantri CK, et al. Coding and non-coding roles of MOCCI (C15ORF48) coordinate to regulate host inflammation and immunity. Nat Commun. 2021;12:2130. 33837217 10.1038/s41467-021-22397-5 PMC8035321

[26] Zhou S, Liu L, Li H, Eilers G, Kuang Y, Shi S, et al. Multipoint targeting of the PI3K/mTOR pathway in mesothelioma. Br J Cancer. 2014;110:2479–88. 24762959 10.1038/bjc.2014.220 PMC4021537

[27] Zauderer MG, Alley EW, Bendell J, Capelletto E, Bauer TM, Callies S, et al. Phase 1 cohort expansion study of LY3023414, a dual PI3K/mTOR inhibitor, in patients with advanced mesothelioma. Invest N Drugs. 2021;39:1081–8. 10.1007/s10637-021-01086-6 PMC8280020 33660194

[28] Singh A, Pruett N, Pahwa R, Mahajan AP, Schrump DS, Hoang CD. MicroRNA-206 suppresses mesothelioma progression via the Ras signaling axis. Mol Ther Nucleic Acids. 2021;24:669–81. 33996251 10.1016/j.omtn.2021.04.001 PMC8093312

[29] Obacz J, Valer JA, Nibhani R, Adams TS, Schupp JC, Veale N, et al. Single-cell transcriptomic analysis of human pleura reveals stromal heterogeneity and informs in vitro models of mesothelioma. Eur Respir J. 2024;63:2300143. 38212075 10.1183/13993003.00143-2023 PMC10809128

[30] The Cancer Genome Atlas Research Network. Integrated genomic analyses of ovarian carcinoma. Nature. 2011;474:609–15. 21720365 10.1038/nature10166 PMC3163504

[31] Drost J, Karthaus WR, Gao D, Driehuis E, Sawyers CL, Chen Y, et al. Organoid culture systems for prostate epithelial and cancer tissue. Nat Protoc. 2016;11:347–58. 26797458 10.1038/nprot.2016.006 PMC4793718

[32] Elyada E, Bolisetty M, Laise P, Flynn WF, Courtois ET, Burkhart RA, et al. Cross-species single-cell analysis of pancreatic ductal adenocarcinoma reveals antigen-presenting cancer-associated fibroblasts. Cancer Discov. 2019;9:1102–23. 31197017 10.1158/2159-8290.CD-19-0094 PMC6727976

[33] Huang H, Wang Z, Zhang Y, Pradhan RN, Ganguly D, Chandra R, et al. Mesothelial cell-derived antigen-presenting cancer-associated fibroblasts induce expansion of regulatory T cells in pancreatic cancer. Cancer Cell. 2022;40:656–73.e7. 35523176 10.1016/j.ccell.2022.04.011 PMC9197998

[34] Sandoval P, Jiménez-Heffernan JA, Rynne-Vidal Á, Pérez-Lozano ML, Gilsanz Á, Ruiz-Carpio V, et al. Carcinoma-associated fibroblasts derive from mesothelial cells via mesothelial-to-mesenchymal transition in peritoneal metastasis. J Pathol. 2013;231:517–31. 24114721 10.1002/path.4281

[35] Gordillo CH, Sandoval P, Muñoz-Hernández P, Pascual-Antón L, López-Cabrera M, Jiménez-Heffernan JA. Mesothelial-to-Mesenchymal Transition Contributes to the Generation of Carcinoma-Associated Fibroblasts in Locally Advanced Primary Colorectal Carcinomas. Cancers. 2020;12:499. 32098058 10.3390/cancers12020499 PMC7072259

[36] Xie Z, Bailey A, Kuleshov MV, Clarke DJB, Evangelista JE, Jenkins SL, et al. Gene Set Knowledge Discovery with Enrichr. Curr Protoc. 2021;1:e90. 33780170 10.1002/cpz1.90 PMC8152575

