# Supplementary material for: Fibroblast-like cells in mesothelioma can derive from tumor cells
Source: Cell Death Differ. 2025 Dec 16;33(6):1203–17. doi: 10.1038/s41418-025-01639-9 (PMC13247067; doi:10.1038/s41418-025-01639-9)
Supplement: Supplementary file 1 — Supplementary figures [file 41418_2025_1639_MOESM1_ESM.pdf]

Fibroblast-like Cells in Mesothelioma Can Derive from Tumor Cells

Running title: Fibroblast-like tumor cells in mesothelioma

Jose M. Garcia-Manteiga[1]*, Eltjona Rrapaj[2,*§], Francesca Caprioglio[2,3]*, Francesco De Marchis[2,3], Andrea Lamarca[3], Liam S. Colley[2], Angelo Carretta[3,4], Daniela Finocchiaro[5], Francesca Mercalli[6], Annamaria Molinario[2], Gianluigi Arrigoni[5], Renzo Boldorini[6], Massimo P. Crippa[2], Rosanna Mezzapelle[2,3,**] and Marco E. Bianchi[2,3,**]

* these authors contributed equally
** correspondence to bianchi.marco@hsr.it and mezzapelle. rosanna@hsr.it

[1] Center for Omics Sciences, IRCCS Ospedale San Raffaele, Milan, Italy
[2] School of Medicine, Vita-Salute San Raffaele University, Milan, Italy
[3] Chromatin Dynamics Unit, Division of Genetics and Cell Biology, IRCCS Ospedale San Raffaele, Milan, Italy
[4] Department of Thoracic Surgery, IRCCS San Raffaele Scientific Institute, Milan, Italy
[5] Department of Pathology, IRCCS Ospedale San Raffaele, Milan, Italy
[6] Department of Health Science, School of Medicine, University of Eastern Piedmont Amedeo Avogadro, Novara, Italy

[§]present address: Department of Cell and Molecular Biology, Karolinska Institutet, Berzelius väg 35, 171 77 Stockholm, Sweden.

SUPPLEMENTRAY FIGURES

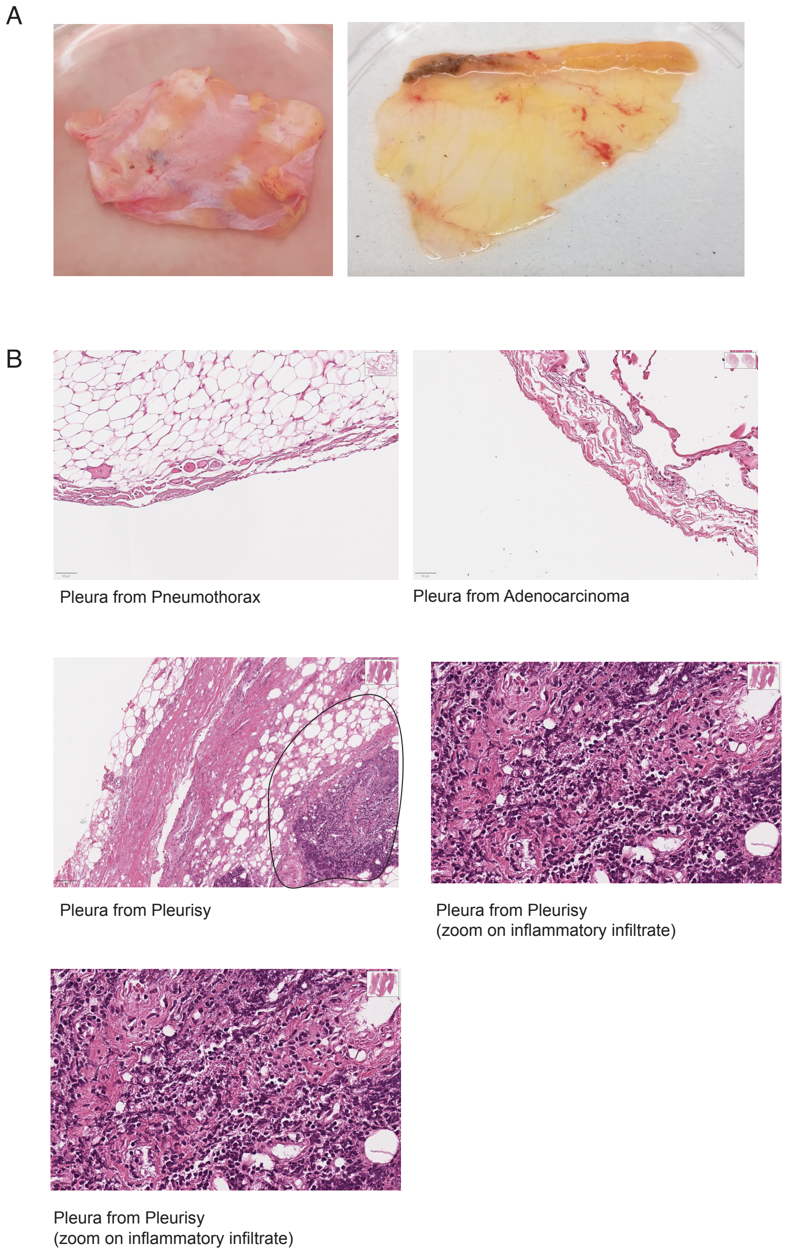

Supplementary Fig. 1. Examples of non-malignant pleura biopsies.

a Macroscopic feature of non-tumor pleura. **b** H&E staining of non-malignant pleura samples. The black circle in the left panel marks the inflammatory infiltrate. Scale bar 500 µm.

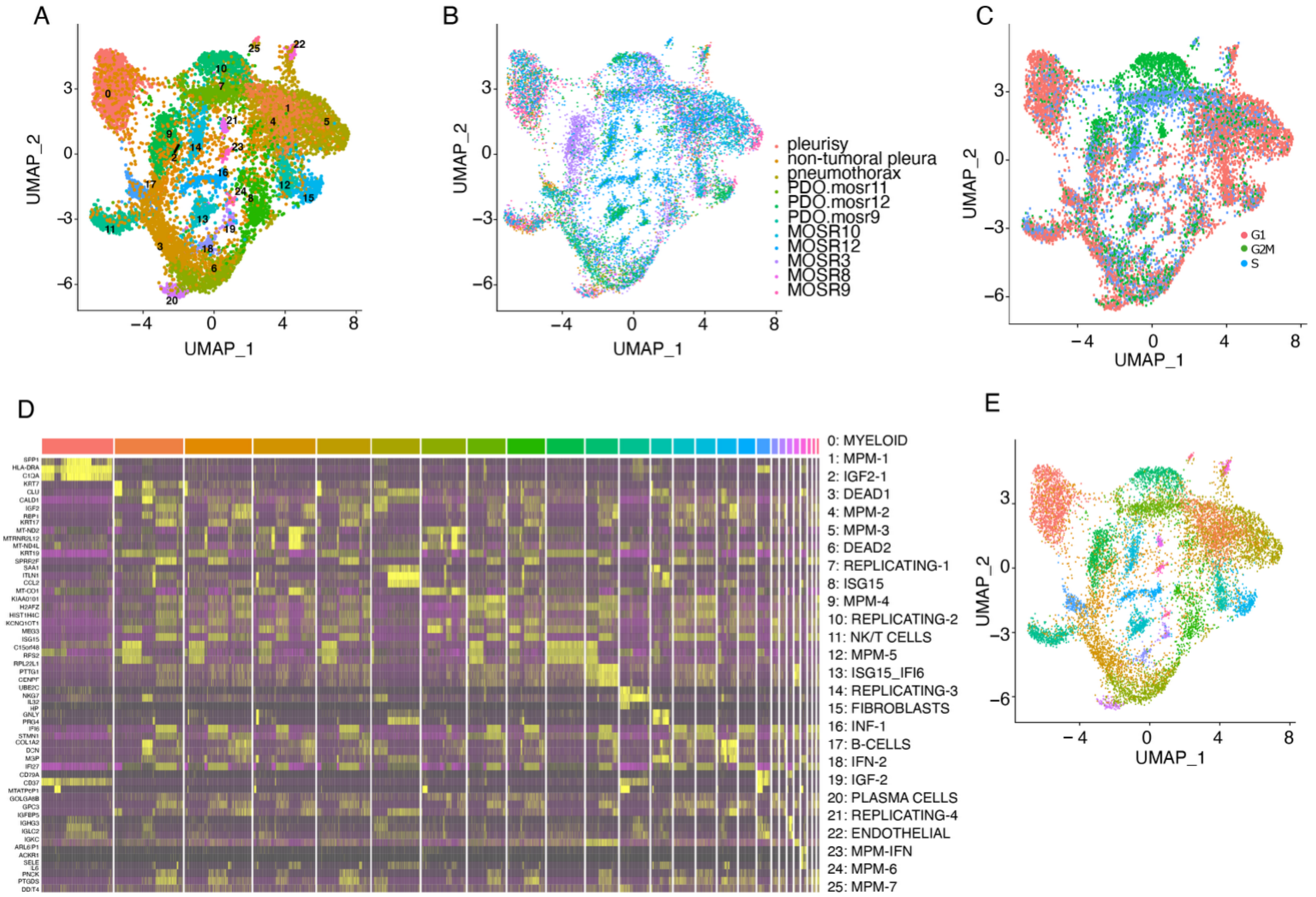

Supplementary Fig. 2. Preliminary analysis of the integration of all samples and all cells.

a UMAP visualization of 26 unbiased cell clusters found using the Louvain method after the integration of the 12,357 cells coming from all 11 samples. Integration was obtained by the Seurat anchoring procedure after scaling and PCA (nPCs=30) using the standard Seurat 3.1 workflow. **b** UMAP visualization of the same cell representation colored by sample origin**. c** Identification of the cell cycle phase of individual cells. **d** Top3 specific markers of all 26 cell clusters found at a resolution of 0.5 by using FindAllMarkers (Wilcoxon test and only.pos = True). **e** Cluster identities assigned with the aid of specific markers and gene signatures (**Supplementary Figure 4**, **Supplementary tables 1,2**) (min.genes =200, min.cells=3). No filter for mitochondrial genes, no filter for max number of genes (doublets), no correction for either of them or for cell cycle was applied.

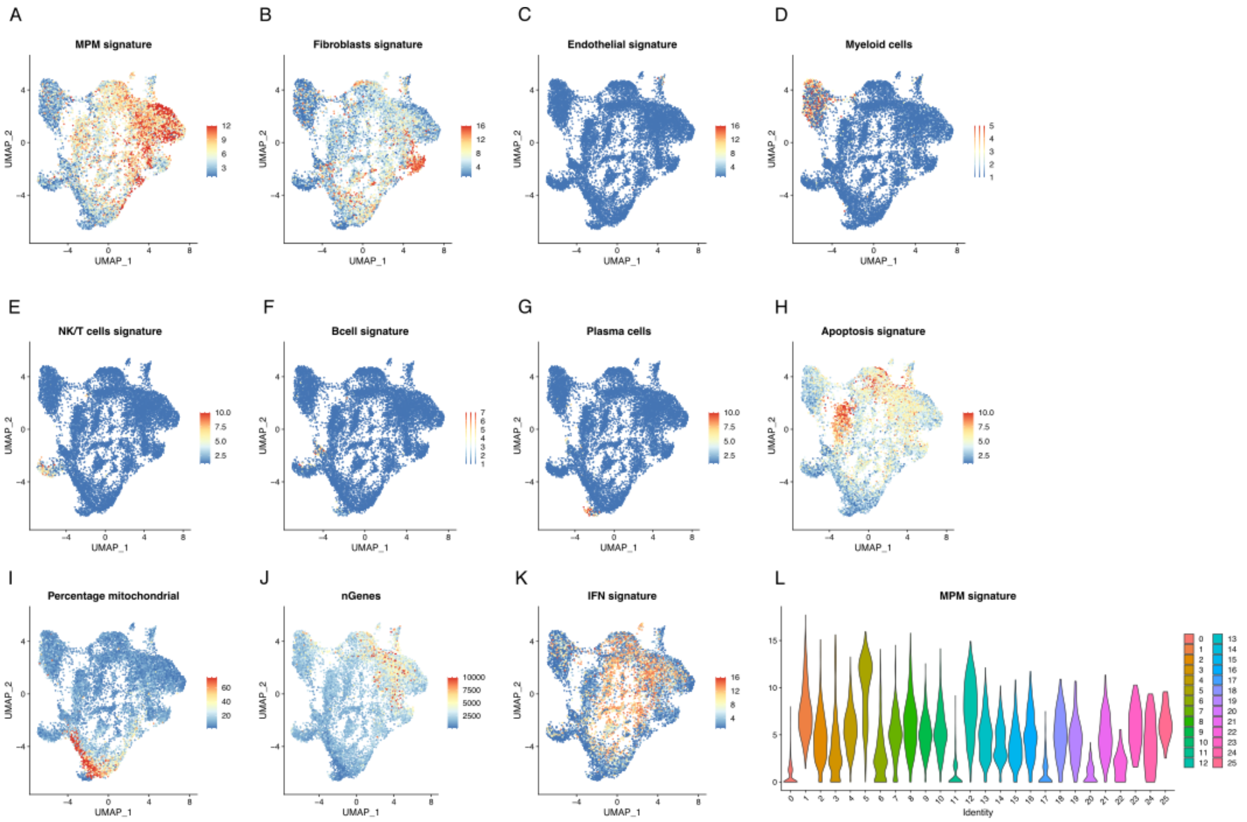

Supplementary Fig. 3. UMAP representation of different gene signatures in the integrated samples.

a PM cluster was characterized by the expression of WT1, KRT7, KRT8, and CALD1. **b** Cancer Associated Fibroblasts were expressing COL1A1, COL1A2, and COL3A1. **c** Endothelial cells express CD34, VWF, SELE, ADAMTS4 and ACKR1. **d** Myeloid cells cluster was identified by the expression of CD14. **e** NK/T cells express CD3G, NKG7 and TRAC. **f** B-cells express CD79A and CD79B. **g** Plasma cells express specifically JCHAIN. **h** A group of cells was identified expressing higher levels of apoptotic genes (Supplementary Table 2). Higher percentage of reads mapping to mitochondrial genes (**i**) and a lower number of expressed genes (**j**) identified a group of dead or suffering cells. **k** Clusters with markers corresponding to IFN response were detected (ISG15, IFI6 and IGF-2), mainly linked to cells coming from biopsy MOSR12. **l** Violin Plot of levels of expression of MPM signature (**Supplementary Table 2**).

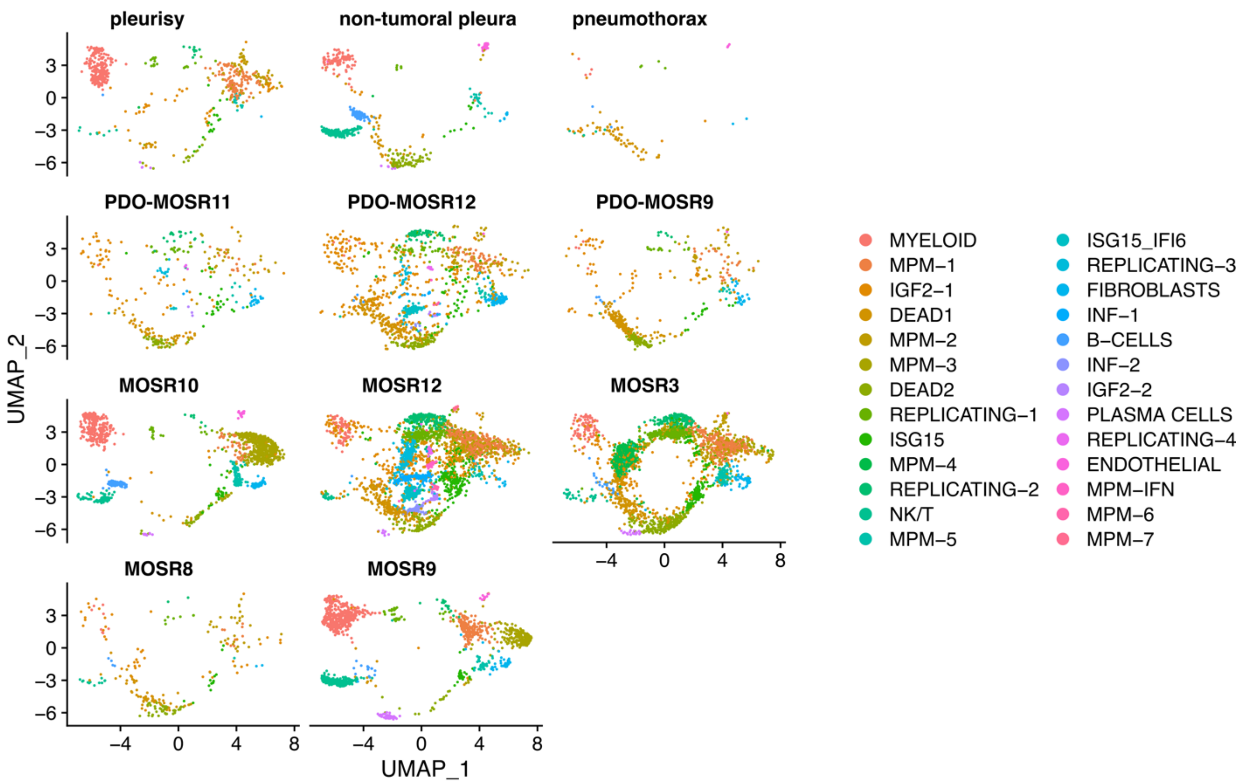

Supplementary Fig. 4. UMAP plot of all samples integrated.

Separate UMAP plots of the eleven different samples in order to appreciate the proportion of the different subpopulations. The IGF-2, ISG15 and other IFN response clusters were mainly found in cells of biopsy MOSR12 and its corresponding PDOs. Dead and low-quality cells were found in all eleven samples.

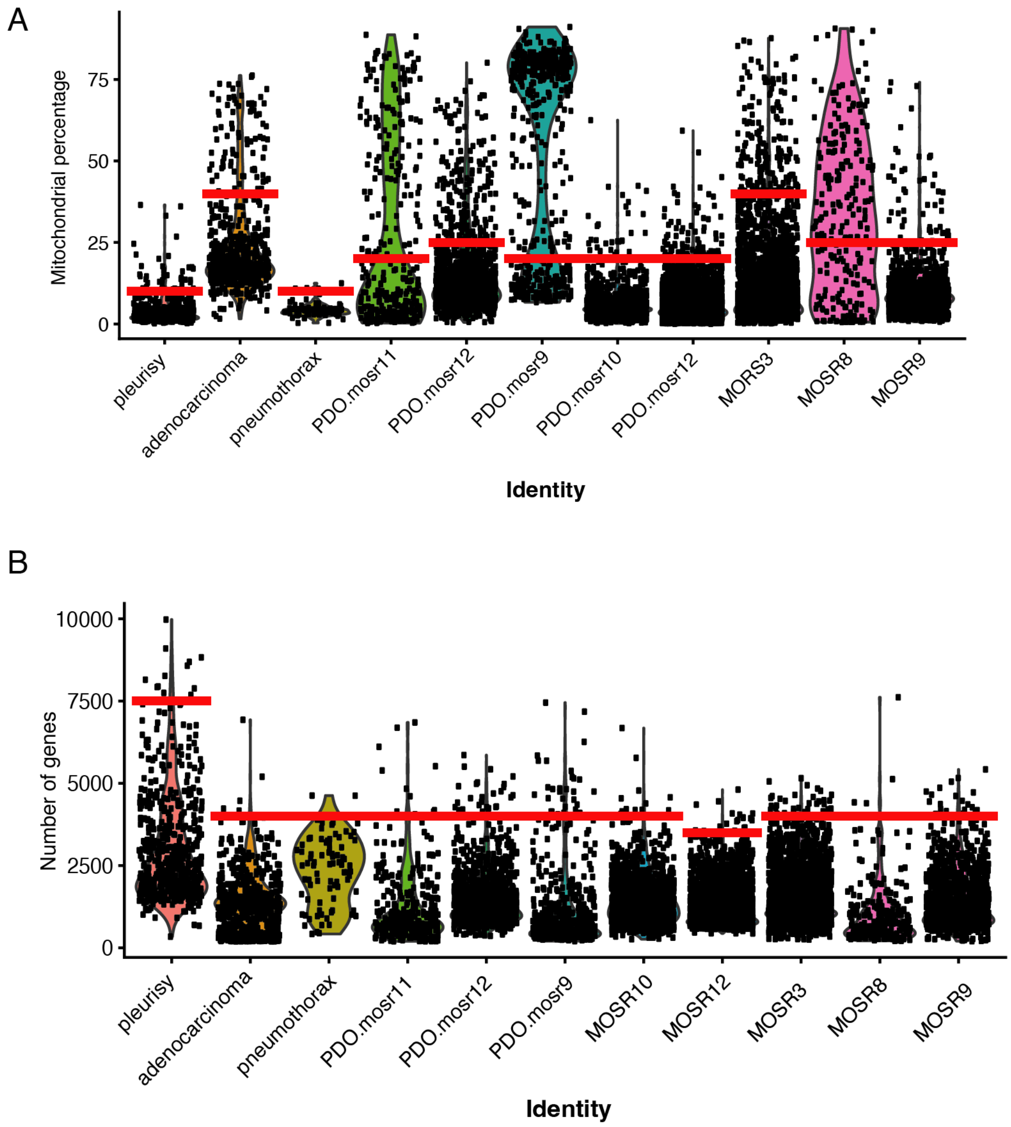

Supplementary Fig. 5. Quality control parameters of cells from all samples.

Violin Plot showing the values of two quality parameters in the eleven samples: number of genes per cell and percentage of reads mapped to mitochondrial genes. The red bar shows the cut offs applied for each sample for mitochondrial percentage **(a)** and max number of genes **(b)** to discard dead/low quality cells and doublets, respectively. Cutoffs were used for each sample independently before applying the anchoring procedure of integration.

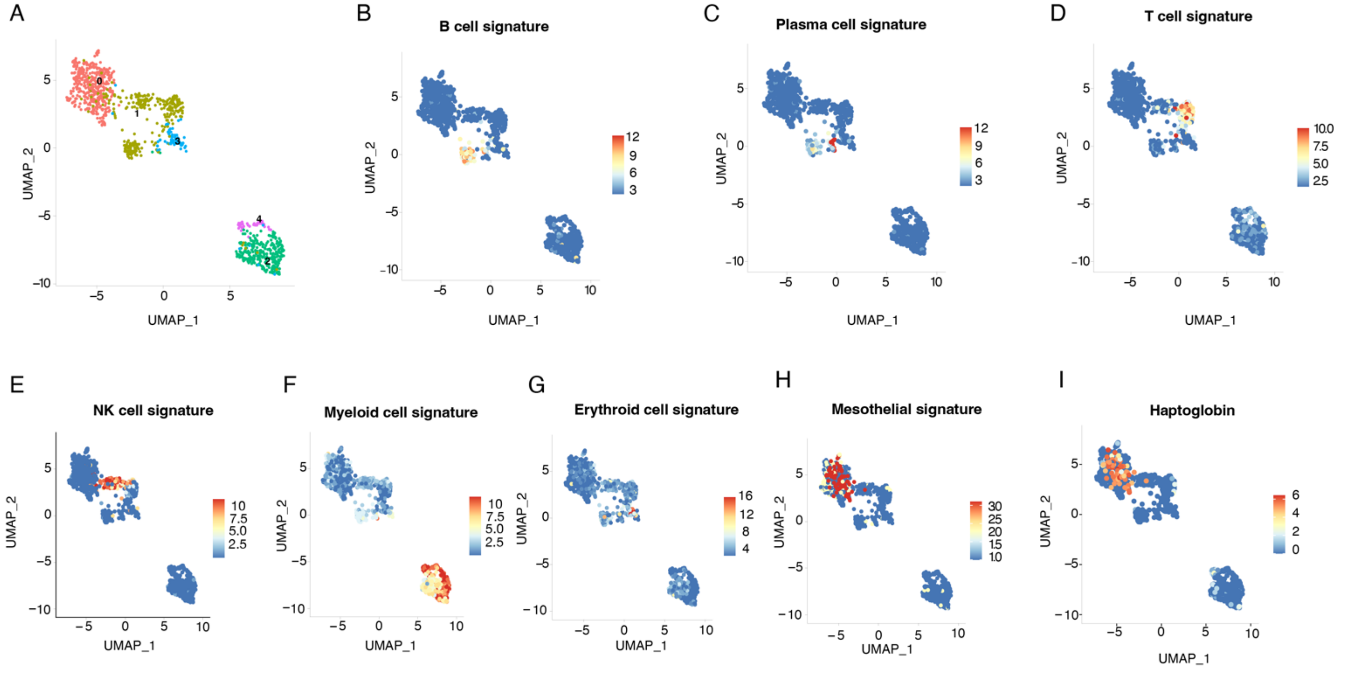

Supplementary Fig. 6. Unbiased clustering of integrated non-malignant pleuras.

a Five clusters were identified by using Seurat standard workflow with a resolution of 0.5. **b-i** Different subclusters were identified by using specific cell gene signatures. found in **Supplementary Table 2**. This information guided the identification of cell types in Figure 2.

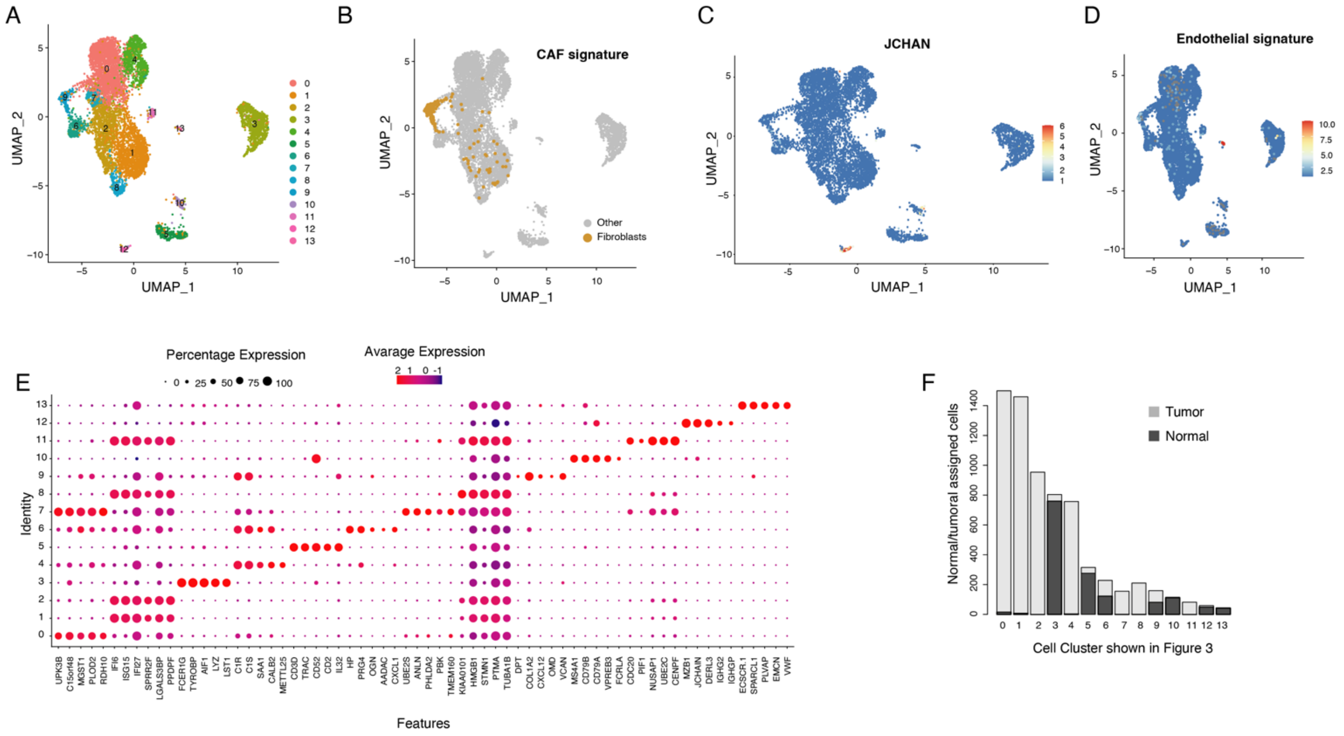

Supplementary Fig. 7. Mesothelial cell identity in PM and non-malignant pleural biopsies.

a UMAP plot of 14 unbiased clusters of integrated PM biopsies after filtering out dead and low-quality cells using a resolution of 0.1. **b** Cancer Associated Fibroblasts identified by the label transfer procedure in Seurat using dataset by Giotti et al. **c** Expression levels of J chain gene identified cluster 12 as plasma cells. **d** Expression levels of endothelial cell signature genes. **E** Dot plot of the top5 cluster specific markers of the clusters in A. **f** Number of non-malignant and tumor cells identified in biopsies based on scRNA-Seq CNV call by Numbat by cluster as in A.

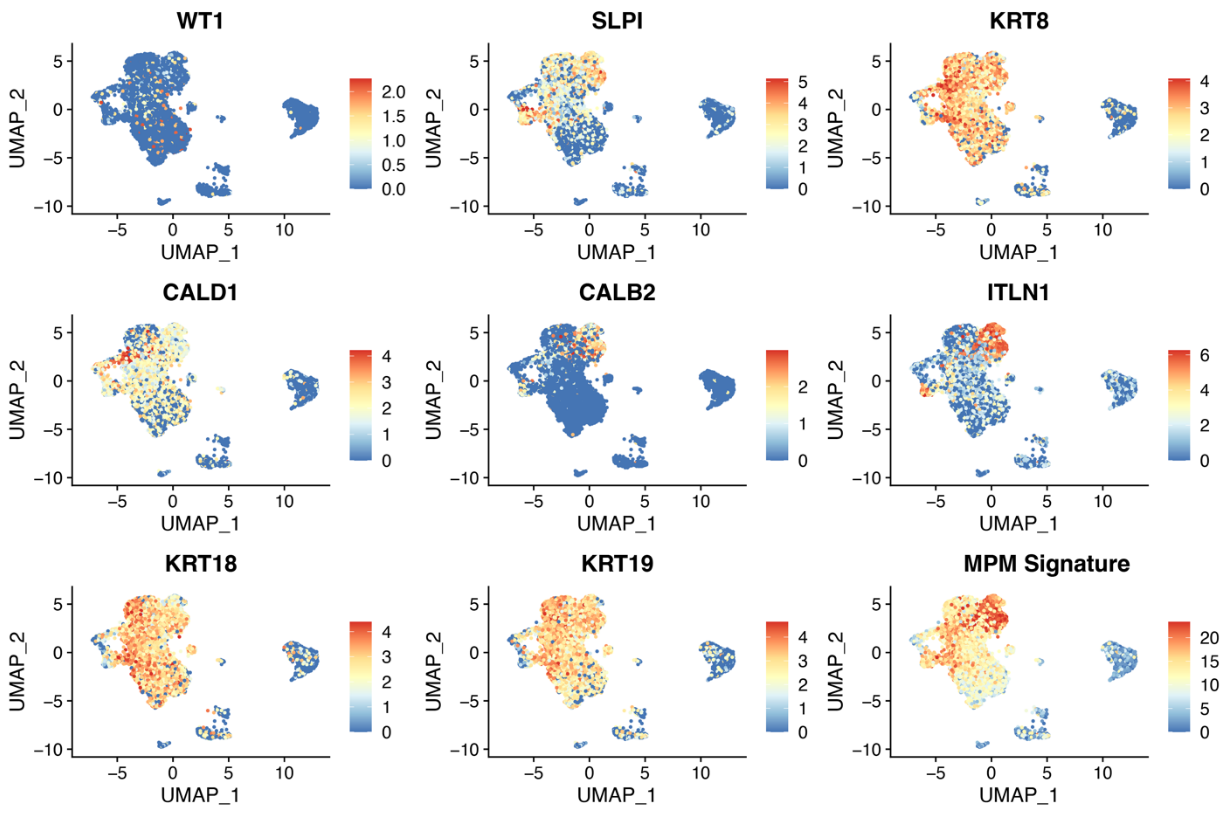

Supplementary Fig. 8. Feature Plot of PM signature and markers.

Expression levels of different mesothelioma markers and a signature comprising all of them (**Supplementary table 2**). Clusters 0, 1, 2, 3, 4, 7, 8 and 11 in Supplementary Figure 7A were assigned to PM cells in Figure 3A based on the expression of MPM signature markers (**Supplementary Tables 2,4**).

A

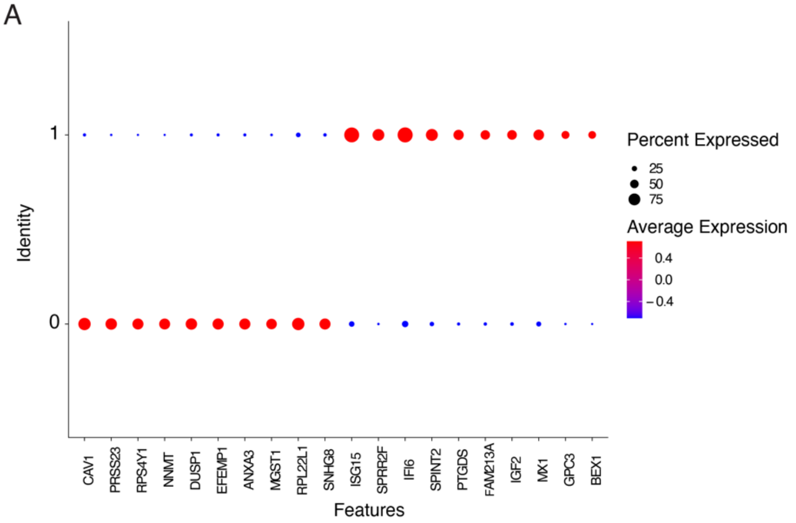

B

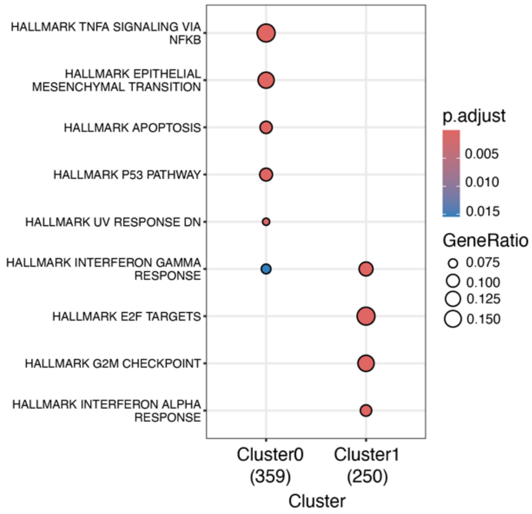

Supplementary Fig. 9. Comparison of clusters comprising mesothelioma and mesothelial cells

a. Top specific markers of cell mesothelial tumor and non-tumor populations in **Fig 4**. **b**. Top5 enriched Hallmark Pathways (2020) using markers for each cluster (logFC > 1, FDR < 0.05).

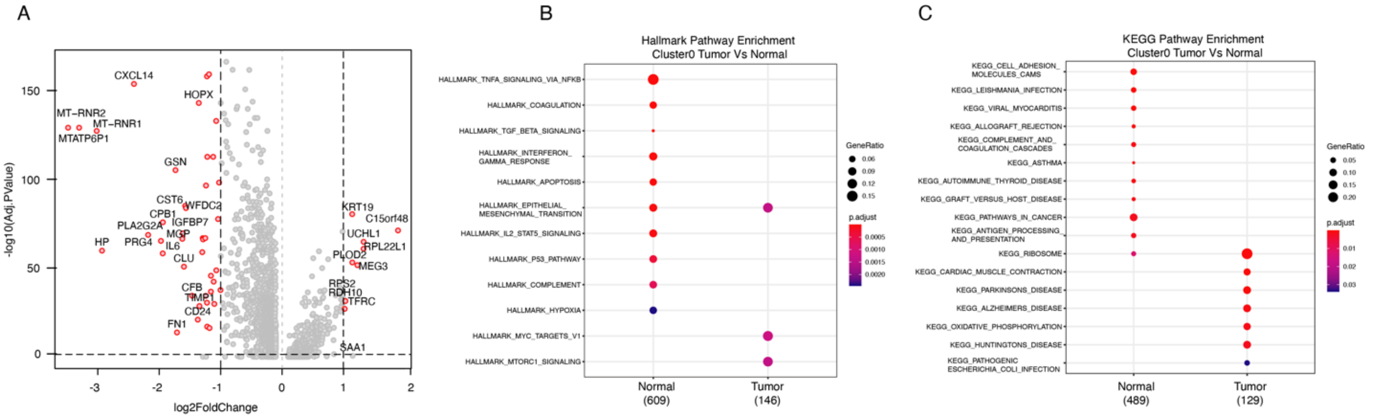

Supplementary Fig. 10. Differences between mesothelioma and mesothelial cells.

a Volcano Plot showing the differentially expressed genes in mesothelioma cells from **Fig. 4** cluster 0 compared to non-malignant mesothelial cells in the same cluster (from tumor and pneumothorax samples) (Supplementary Table 6). Differentially expressed genes were found using *FindClusters*; FDR<0.05, log2FC > 1. Functional enrichment using genes with FDR < 0.05 and a log2FC > 0.5 within the Hallmark MSigDB pathway database **b** or KEGG Pathways **c** by using *ClusterProfiler* R Package. The top 10 pathways for each comparison are shown.

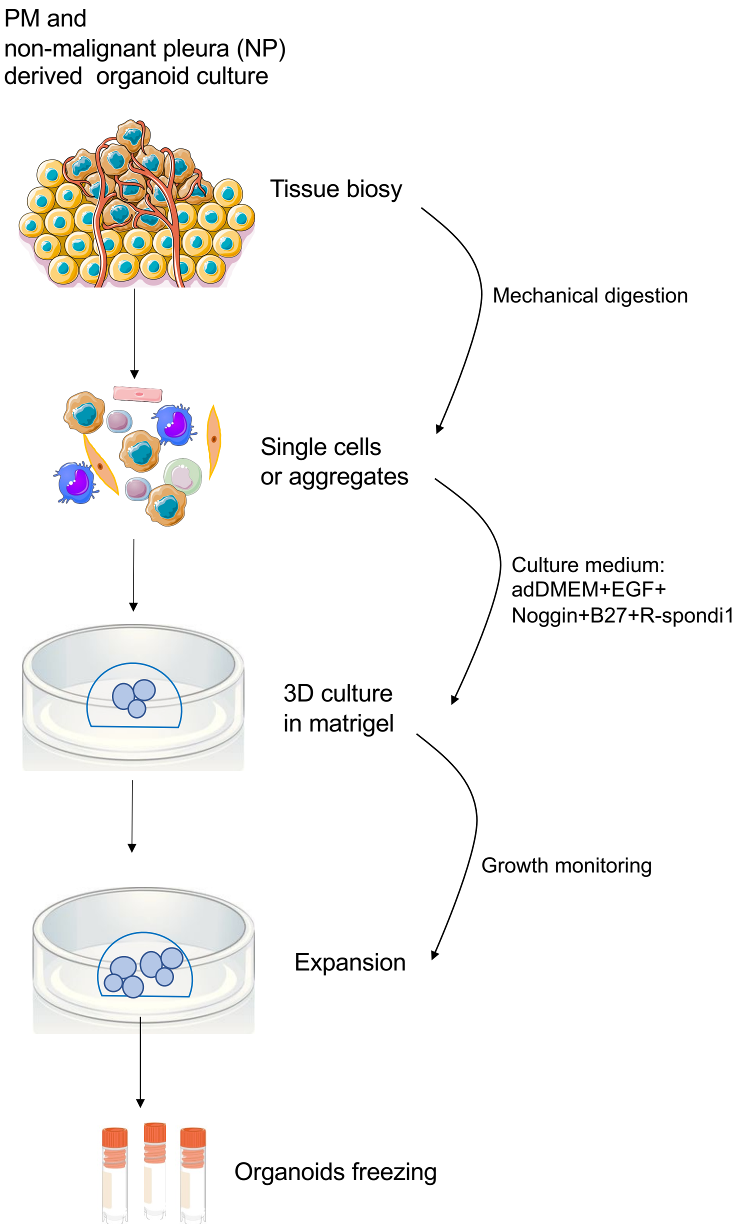

Supplementary Fig. 11. Procedure for the generation of PDOs.
Schematic representation of the procedure for establishing organoids from normal pleura (NP) and malignant pleural mesothelioma (PM) biopsies. Biopsies are mechanically minced to level of cell aggregates and then cultured in Matrigel supplemented with culture medium (see Materials section). Expanded organoids can be frozen and stored in liquid nitrogen.

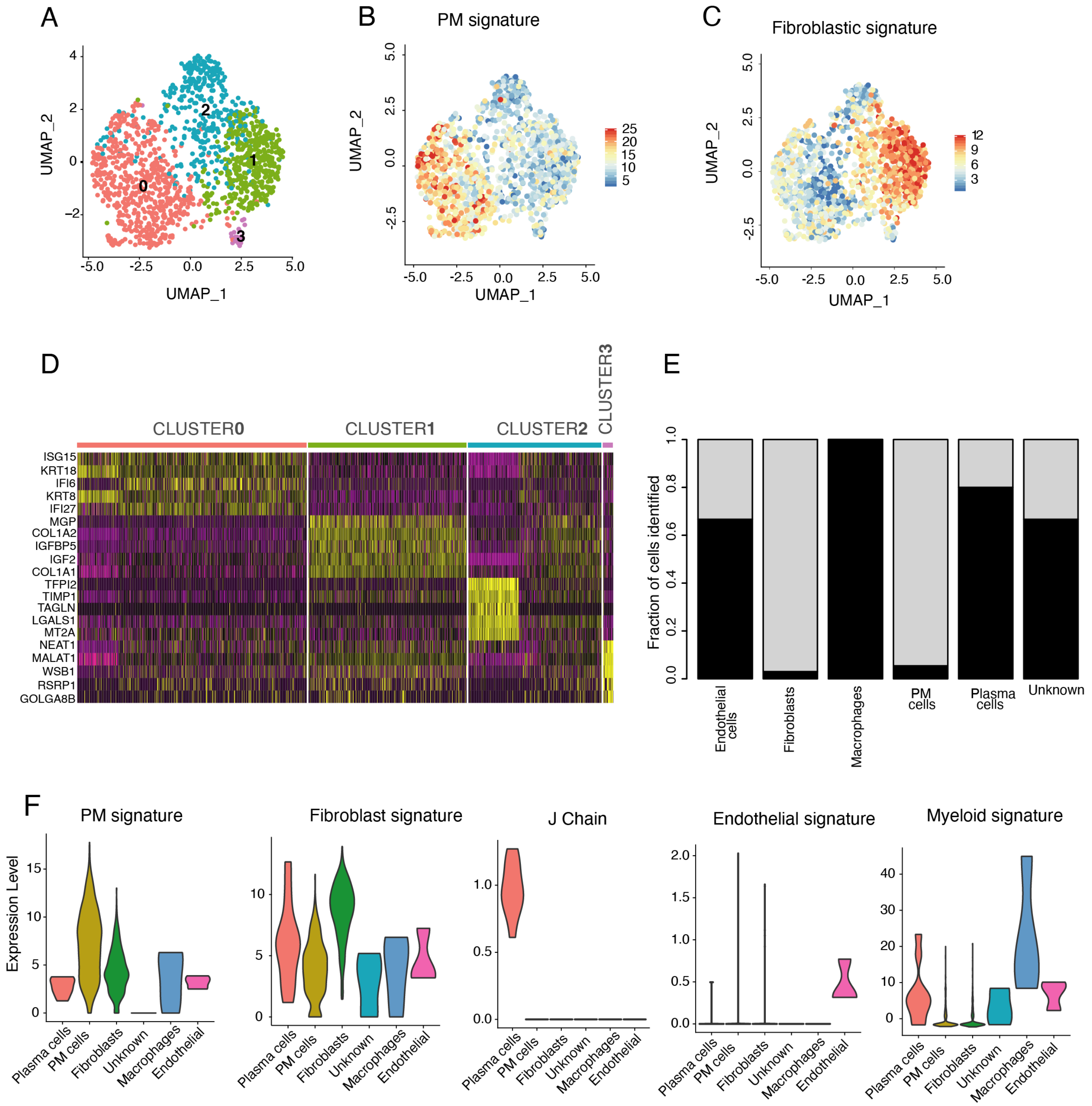
A
UMAP_2
UMAP_1
0
1
2
3
B
PM signature
C
Fibroblastic signature
D
CLUSTER0
CLUSTER1
CLUSTER2
CLUSTER3
ISG15
KRT18
IFI6
KRT8
IFI27
MGP
COL1A2
IGFBP5
IGF2
COL1A1
TFPI2
TIMP1
TAGLN
LGALS1
MT2A
NEAT1
MALAT1
WSB1
RSRP1
GOLGA8B
E
Fraction of cells identified
Endothelial cells
Fibroblasts
Macrophages
PM cells
Plasma cells
Unknown
F
PM signature
Fibroblast signature
J Chain
Endothelial signature
Myeloid signature
Expression Level
Plasma cells
PM cells
Fibroblasts
Unknown
Macrophages
Endothelial

Supplementary Fig. 12. Characterization of cells in PDOs.

a UMAP of cells from PDOs unbiased clustering. While cluster 1 mainly expressed collagens and fibroblastic markers, Cluster 2 showed expression of markers of EMT such as TIMP1, TAGLN and TFPI2. **b** Expression of an PM signature in PDO cells identified cluster 0 as PM cells with IFN signaling. **c** Expression of Fibroblastic signature in PDO cells identified cluster 1 as fibroblasts. **d** Heatmap with top five markers of unbiased clusters in panel A. Cluster2 cells contained cells showing the expression of markers of Epithelial to Mesenchymal Transition such as TIMP1, TAGLN and TFPI2 and mainly derived from PDO-MOSR9. **e** Barplot of Normal/Tumor proportions for each cell type in PDOs in **Fig. 6A,** using single cell CNV analysis by Numbat. The results confirm the tumoral nature of cells identified as fibroblasts. **f** Violin plots of the expression of the different signatures (**Supplementary Table 2**) in cells shown in Fig.6A.

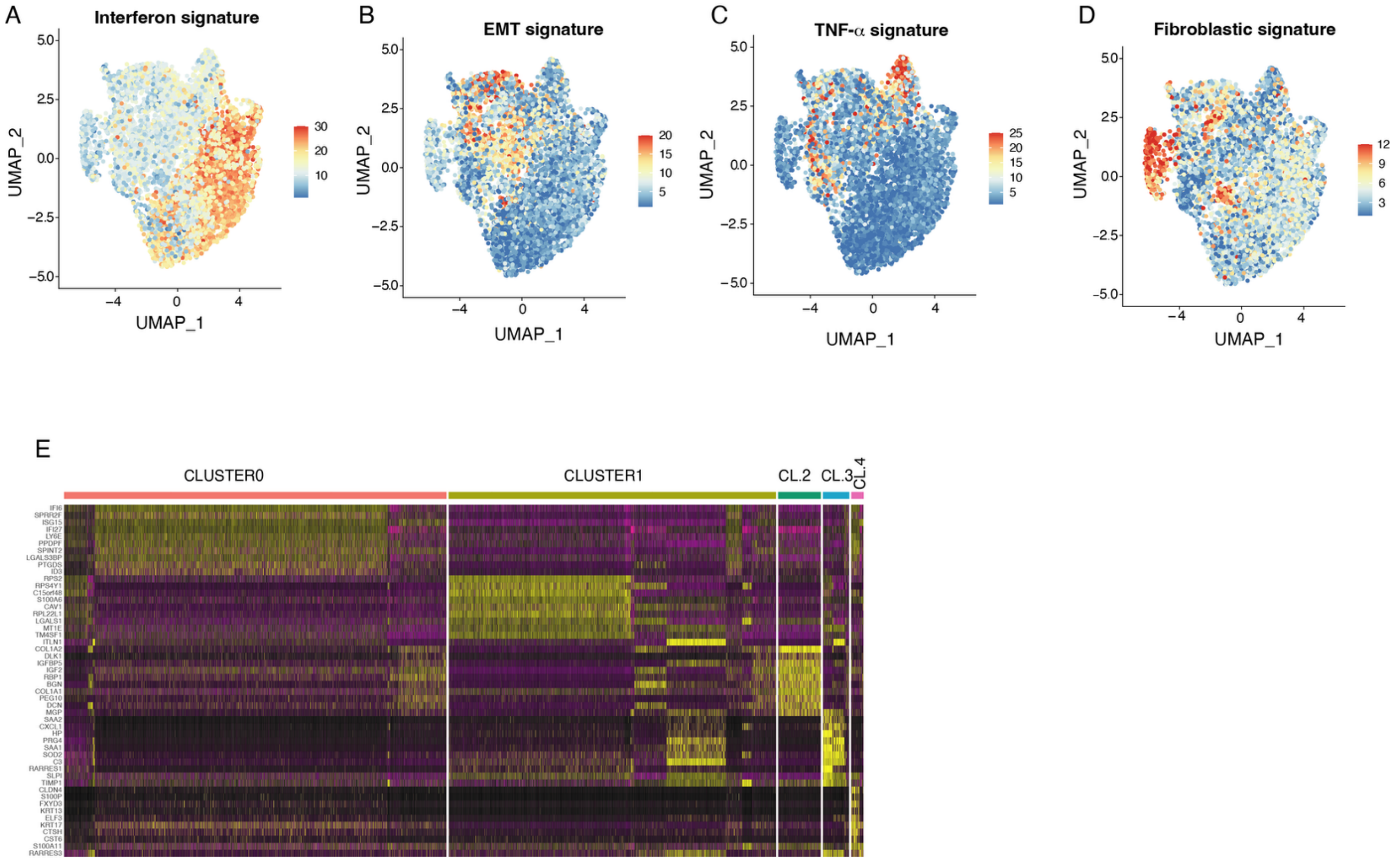

Supplementary Fig. 13. Integrated analysis of mesothelial cells from PM biopsies and PDOs.

a-d UMAP plot representation of the expression of gene signatures as in **Fig. 6c**. **e** Heatmap plot of levels of expression of top10 specific markers of clusters in **Fig.6c**.

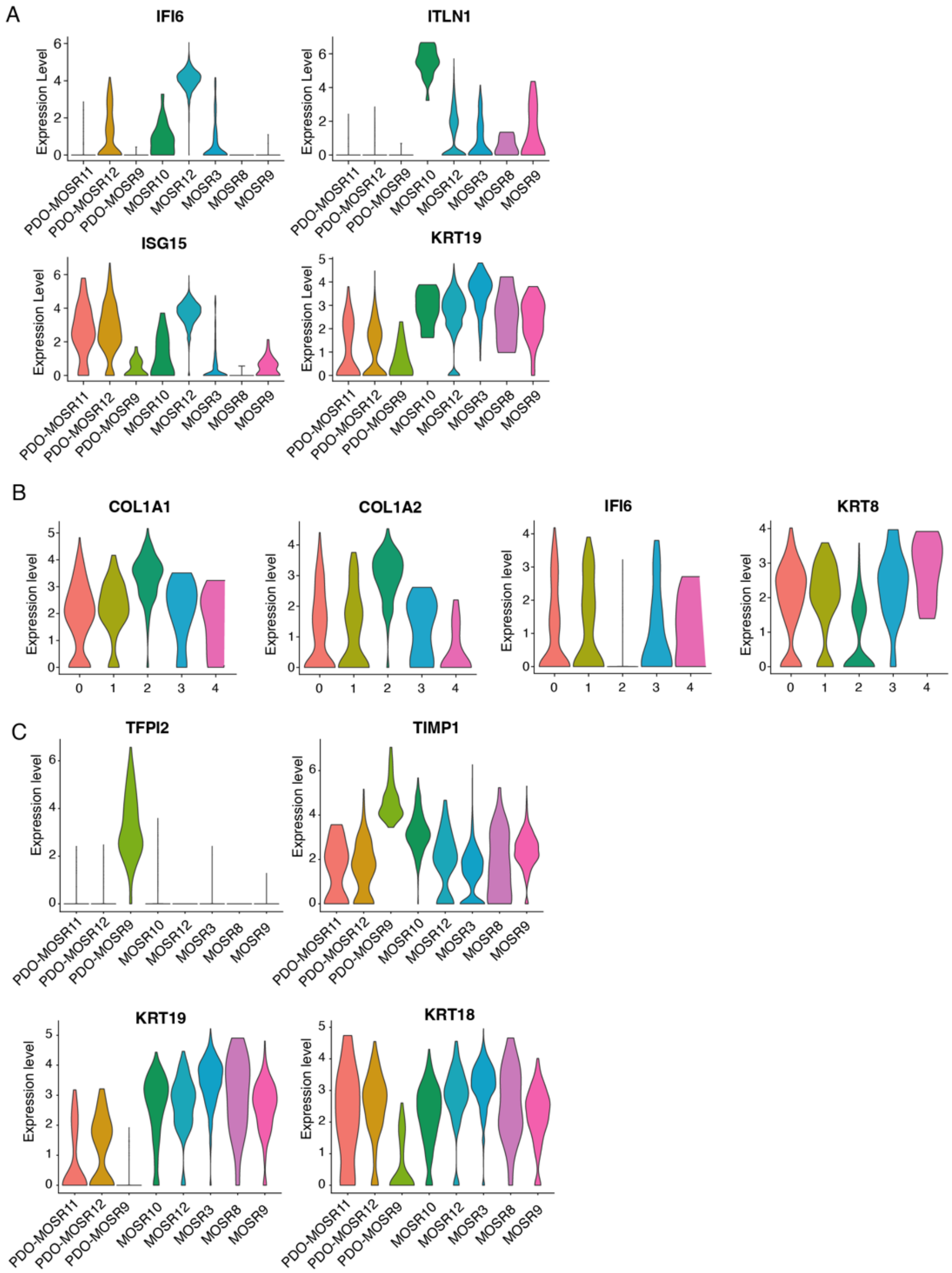

Supplementary Fig. 14. Violin plots showing the expression of selected genes differentially expressed between PDOs and PM biopsies.

a Violin Plots of expression levels for selected genes down regulated in PDOs in cluster 0 from tumor12 with respect to the biopsy. **b** Expression levels of COL1A1, COL1A2 in the different clusters, as examples of upregulated genes, and IFI6 and KRT8, as examples of downregulated genes, in the comparison between cluster 2 vs cluster 0 cells in PDO-MSR12. **c** Expression levels of TFPI2 and TIP1 in the different samples, as examples of upregulated genes, and KRT19 and KRT18, as examples of downregulated genes, in cells from cluster 1 in organoids vs in the biopsy of the same patient (tumor9).

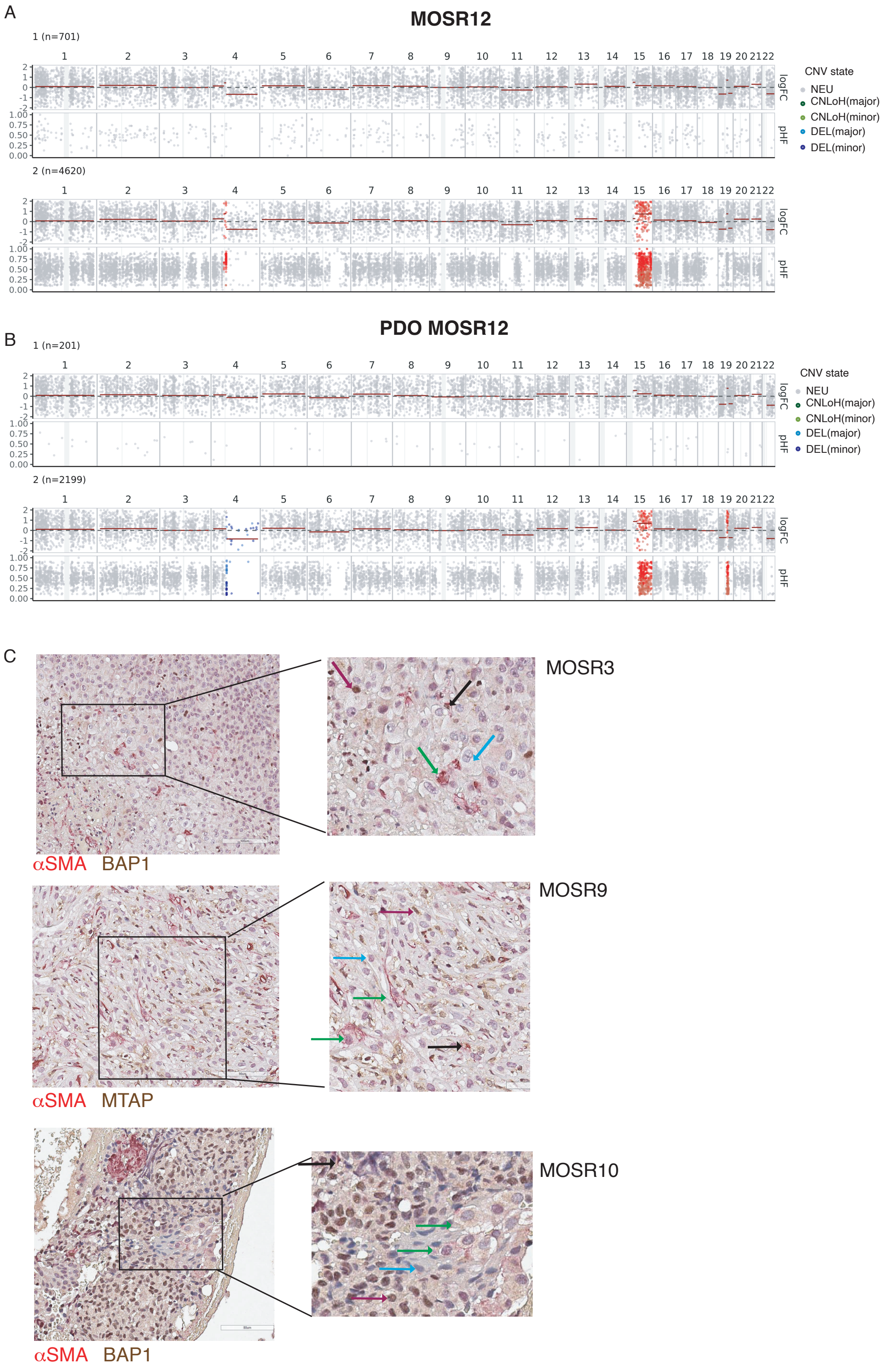
A
MOSR12
1 (n=701)
2 (n=4620)
logFC
pHF
CNV state
NEU
CNLoH(major)
CNLoH(minor)
DEL(major)
DEL(minor)
B
PDO MOSR12
1 (n=201)
2 (n=2199)
C
MOSR3
αSMA BAP1
MOSR9
αSMA MTAP
MOSR10
αSMA BAP1

Supplementary Fig. 15 Single-cell CNV detection in biopsy MOSR12 and immunohistochemistry enable the identification of cancer-derived fibroblasts (CDFs)

a-b CNV profile of MOSR12 and PDO MOSR12 cells; log(FC), log expression fold-change; pHF, parental haplotype frequency; NEU, neutral; CNLoH, copy-neutral Loss of Heterozigosity; DEL, deletion; AMP, amplification. Gray vertical bars represent centromeres and gap regions. The upper profile(1) is the one found in non-tumoral cells, without CNVs, the lower profile (2) is of a single clone of mesothelioma cells with CNVs. The number of cells within each profile is shown in parenthesis. **c** Immunohistochemistry of the MOSR3 and MOSR12 biopsies. αSMA is red and cytoplasmic, BAP1 is brown and mostly nuclear. The blue arrow points to one double-negative cell (mesothelioma tumor cell), the brown arrow to one αSMA-negative BAP1-positive cell (infiltrating immune cell), the black arrow to one double-positive cell (vascular smooth cell or pericyte) and the green arrows to two αSMA- positive BAP1-negative cells (CDFs).

Supplementary Table 1. Clinical and pathological features of additional pleural mesothelioma patients.

Patient	Gender	Age at diagnosis	Histotype	History of asbestos exposure	CK 5	Calretinin	MTAP
MOSR85	M	79	Sarcomatoid	Likely	+	+	-
MOSR86	F	66	Biphasic	NA	+	+	+
MOMC1	F	79	Sarcomatoid	Yes	NA	+	-
MOMC2	M	61	Sarcomatoid	Yes	-	+	-

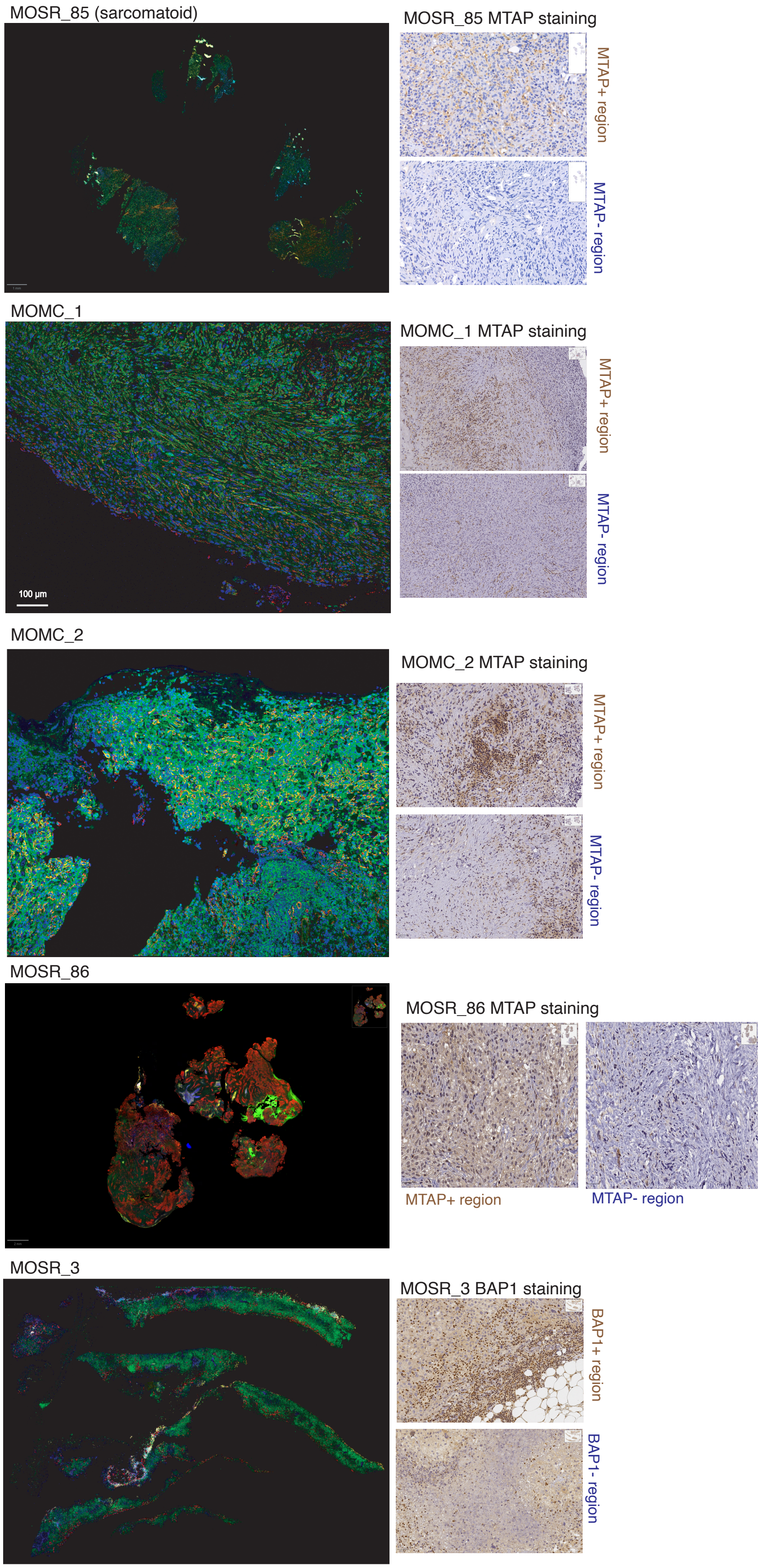
MOSR_85 (sarcomatoid)
MOSR_85 MTAP staining
MTAP+ region
MTAP- region
MOMC_1
100 µm
MOMC_1 MTAP staining
MTAP+ region
MTAP- region
MOMC_2
MOMC_2 MTAP staining
MTAP+ region
MTAP- region
MOSR_86
MOSR_86 MTAP staining
MTAP+ region
MTAP- region
MOSR_3
MOSR_3 BAP1 staining
BAP1+ region
BAP1- region

Supplementary Fig. 16 Multiplex immunohistochemistry of an additional cohort of 5 PM samples.

Left panel: Rapresentative images of multiplex IHC (MTAP and BAP1 are in green; CD45 is in yellow; αSMA is in red; nuclei are in blue)**.** Scale bar: MOSR_85: 1mm; MOMC_1: 100μm; MOMC_2: 100μm; MOSR_85and MOSR_3: 2mm. **Right panel**: MTAP or BAP1 IHC staining. Magnification 20X.
